# Ultrafast dynamics of formation and autodetachment of a dipole-bound state in an open-shell π-stacked dimer anion†
†Electronic supplementary information (ESI) available: Experimental and theoretical methods; angle-resolved imaging data; details of global fitting procedure; CASSCF orbitals; resonance wavefunction characters; details of conical intersections; selected illustrations of carbonyl wagging modes; all frequency-resolved spectra. See DOI: 10.1039/c6sc01062h


**DOI:** 10.1039/c6sc01062h

**Published:** 2016-05-04

**Authors:** James N. Bull, Christopher W. West, Jan R. R. Verlet

**Affiliations:** a Department of Chemistry , Durham University , South Road , Durham DH1 3LE , UK . Email: j.r.r.verlet@durham.ac.uk

## Abstract

Isolated π-stacked dimer radical anions present the simplest model of an excess electron in a π-stacked environment. Here, frequency-, angle-, and time-resolved photoelectron imaging together with electronic structure calculations have been used to characterise the π-stacked coenzyme Q_0_ dimer radical anion and its exited state dynamics. In the ground electronic state, the excess electron is localised on one monomer with a planar *para*-quinone ring, which is solvated by the second monomer in which carbonyl groups are bent out of the *para*-quinone ring plane. Through the π-stacking interaction, the dimer anion exhibits a number of charge-transfer (intermolecular) valence-localised resonances situated in the detachment continuum that undergo efficient internal conversion to a cluster dipole-bound state (DBS) on a ∼60 fs timescale. In turn, the DBS undergoes vibration-mediated autodetachment on a 2.0 ± 0.2 ps timescale. Experimental vibrational structure and supporting calculations assign the intermolecular dynamics to be facilitated by vibrational wagging modes of the carbonyl groups on the non-planar monomer. At photon energies ∼0.6–1.0 eV above the detachment threshold, a competition between photoexcitation of an intermolecular resonance leading to the DBS, and photoexcitation of an intramolecular resonance leading to monomer-like dynamics further illustrates the π-stacking specific dynamics. Overall, this study provides the first direct observation of both internal conversion of resonances into a DBS, and characterisation of a vibration-mediated autodetachment in real-time.

## Abstract 2

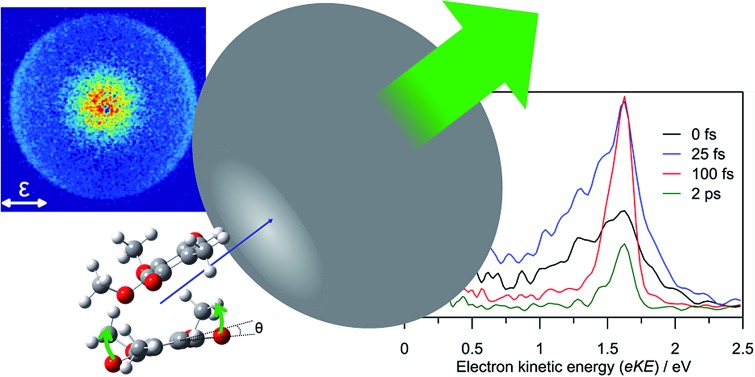
Formation and mode-specific autodetachment from a dipole-bound state in a radical anion dimer is observed in the frequency and time-domains.

## Introduction

The interplay between π-stacking and hydrogen-bonding interactions in large anionic systems is central to a range of physical processes in chemistry, biology and technology. These interactions govern, for example, anion-recognition processes in supramolecular assemblies,^1^,^2^ protein structure and function,^3^,^4^ and solution-phase anion aggregation.^5^,^6^ Excited states of these complexes are important in molecular electronics,^7^–^11^ may be involved in facilitating electron transfer in biological systems,^12^–^14^ and play a key role in the interaction of low energy ballistic electrons with DNA.^15^–^20^ However, gaining a detailed molecular level understanding of anion excited states and their non-adiabatic dynamics is generally hampered by the complex nature and environment of such systems. It is therefore appropriate to consider small π-stacked aggregates as simple models. The gas phase offers an idealised environment in which the desired intra- and intermolecular dynamics can be probed without the added complexity of surroundings. To date, most studies of open-shell π-stacked anion systems have focused around characterising the localised *vs.* delocalised character of ground and excited states.^21^–^27^ However, the dynamics and timescales available to an excess electron in electronically excited states of a prototypical π-stacked system, or the competition between localised (intramolecular) and delocalised (intermolecular) dynamics, have not been experimentally characterised despite their importance in determining their photo-chemistry and physics.

**Fig. 1:**
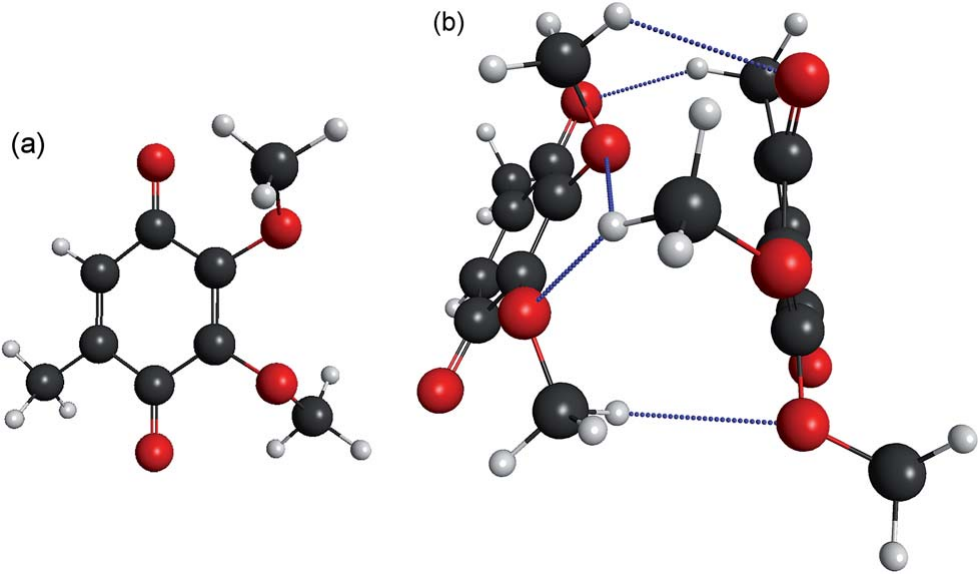
Calculated minimum energy structures for (a) CQ_0_ monomer; (b) equilibrium geometry of π-stacked (CQ_0_)_2_^–^, adopting a distorted sandwich geometry. The excess electron is localised on the left (planar) monomer in the ground electronic state. The right (non-planar) monomer has the carbonyl (C=O) groups bent out of the ring plane by ∼10°. Key: charcoal – carbon; red – oxygen; white – hydrogen; hydrogen bonds – blue dashed.

Here, we consider the coenzyme Q_0_ dimer radical anion, (CQ_0_)_2_^–^, the calculated minimum energy structure of which is shown in Fig. 1. (CQ_0_)_2_^–^ is a useful prototype system because of the strong gas phase stability of one conformer, the well-understood dynamics of the isolated monomer radical anion, CQ_0_^–^,^28^ and its ability to support a cluster dipole-bound state. More generally, *para*-quinones are often considered as prototypical electron acceptors due to their ubiquity in biological and technological electron transfer systems.^29^–^32^ CQ_0_ is the smallest member in the coenzyme-Q series, where the subscript in CQ_0_ refers to the length of an isoprenyl tail attached to the *para*-quinone ring that serves to enhance lipid miscibility. It is thought that the synergetic co-operation of hydrogen bonding and π-stacking of the *para*-quinone ring in biological systems play important roles in facilitating electron transfer.^12^,^33^–^35^ Anionic excited states of *para*-quinones have been proposed as possible bypasses of the inverted Marcus region,^36^–^40^ thus providing a more efficient electron transfer route.

Recent gas phase studies on a series of monomer *para*-quinone radical anions have shown that photoexcitation cross-sections to quasi-bound π*-resonances can be larger than direct photodetachment.^28^,^40^–^42^ These resonances are valence-localised excited states that are embedded in the detachment continuum with inherent autodetachment lifetimes ranging from tens to hundreds of femtoseconds.^43^–^46^ Despite their transient existence, internal conversion can compete with prompt autodetachment.^28^,^40^–^42^,^47^ Resonances can generally be divided into two types: shape resonances, in which the extra electron occupies an unfilled valence orbital of the neutral ground electronic state configuration (∼10^–14^ s lifetimes); and Feshbach resonances, in which the corresponding neutral core is predominately in an electronically excited state (typically 10^–14^ to 10^–12^ s lifetimes). We have recently shown that anion photoelectron (PE) imaging is ideally suited to probe the dynamics of anion resonances,^28^,^40^–^42^,^48^,^49^ allowing identification of signatures from: direct photodetachment into the continuum; prompt autodetachment from a photoexcited resonance; delayed autodetachment from an excited state following internal conversion or extensive nuclear motion; and thermionic emission. Because the spectral contributions from direct photodetachment and prompt autodetachment cannot be easily resolved, they are jointly labelled as prompt detachment.

Neutral molecules or clusters, such as (CQ_0_)_2_, with an electric dipole moment, |***μ***| > 2.5 D, can support a dipole-bound state (DBS),^46^,^50^ in which an excess electron is weakly bound (10–100's meV) in a highly diffuse (non-valence-localised) orbital located at the positive end of ***μ***. Due to the diffuse nature of a DBS, the direct photoexcitation cross-section from a valence-bound state is usually very small. In contrast, photodetachment cross-sections of a DBS can be very large and increase with decreasing photon energy.^51^ DBSs are believed to play an important role in the formation of anions in the interstellar medium and low energy electron capture biochemical systems such as DNA.^15^–^20^,^46^,^52^,^53^

Here, a combined frequency-, angle-, and time-resolved photoelectron imaging (FAT-PI)^41^ and electronic structure study on the spectroscopy and dynamics of excited states of π-stacked (CQ_0_)_2_^–^ is presented. The frequency- and angle-resolved dimensions involve recording single-photon PE images (spectra) at many different photon energies (*hν*) to identify trends and fingerprints of resonances and their associated dynamics. A selected resonance can then be photoexcited and the resulting dynamics monitored in real-time using time-resolved PE imaging. Supporting *ab initio* calculations using multi-state XMCQDPT2 theory with a large CASSCF reference space allow clear assignment of the experimental dynamics.^54^ Complete experimental and theoretical details are given in the ESI.† We show that the excess electron in the ground electronic state exists as a localised charge on one monomer. Photoexcitation of a selected resonance at *hν* = 3.10 eV with high intermolecular charge-transfer character leads to the formation of a DBS on a ∼60 fs timescale, which then undergoes vibration-mediated autodetachment on a 2.0 ± 0.2 ps timescale. This determination represents the first direct observation of the conversion of above-threshold valence-localised population to a DBS, and the first real-time characterisation of vibration-mediated autodetachment of a DBS. At slightly higher *hν*, a competition between non-adiabatic dynamics leading to the DBS and autodetachment dynamics has been observed. The competition is assigned to the interplay between dimer and monomer-like dynamics, and further highlights the role of π-stacking in influencing the excited state dynamics.

## Results and analysis

### Frequency-resolved photoelectron imaging

**Fig. 2:**
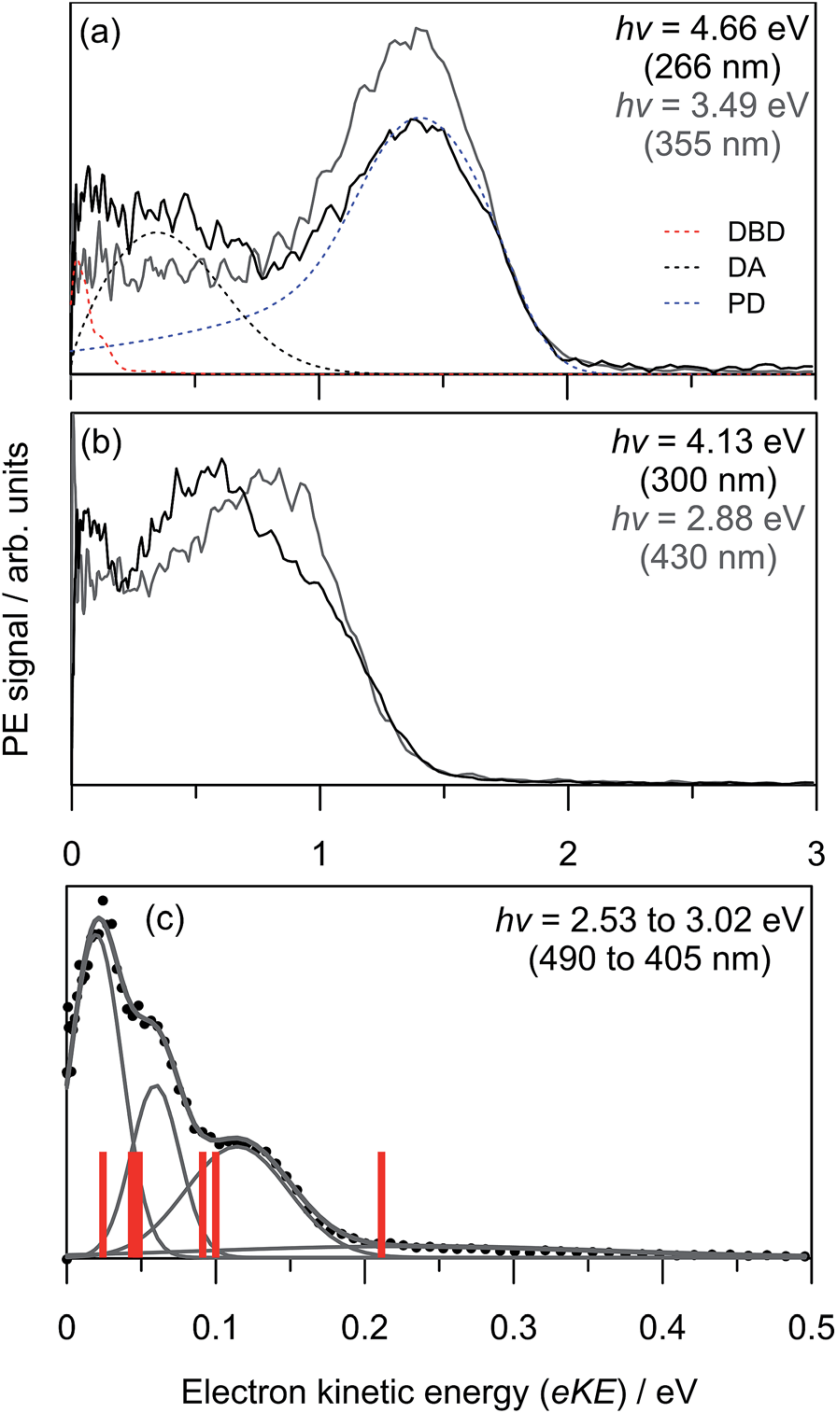
Example (CQ_0_)_2_^–^ photoelectron spectra (black) and CQ_0_^–^ (grey) from ref. 28 at three *hν*: (a) 4.66 eV; (b) 4.13 eV; (c) average between 2.53 eV and 3.02 eV. Included in (a) are the contributions from the three global-fit channels. The red bars in (c) are calculated carbonyl wagging modes localised on the non-planar monomer.

Three example PE spectra of (CQ_0_)_2_^–^ are shown in Fig. 2, recorded at *hν*: (a) 4.66 eV (266 nm); (b) 4.13 eV (300 nm); and (c) the average of 2.53 eV (490 nm) to 3.02 eV (405 nm). The 4.66 eV and 4.13 eV spectra are also compared with selected PE spectra of CQ_0_^–^ that extend to the same maximum in electron kinetic energy (eKE).^28^ In both cases there is a difference in the adiabatic detachment energy (ADE) between CQ_0_^–^ and (CQ_0_)_2_^–^ of ∼1.2 eV. The spectra for (CQ_0_)_2_^–^ and CQ_0_^–^ broadly exhibit similar spectral features, except the dimer generally shows an increased yield of low-eKE electrons. The PE spectrum in Fig. 2(c) is the average of a number of spectra at low *hν*, all of which are identical within noise. These spectra show three (perhaps four) reproducible partially resolved features that have the appearance of vibrational structure. It is remarkable that vibrational-like structure is discernible for such a large molecular system at 300 K, and implies a mode-specific detachment process.

**Fig. 3:**
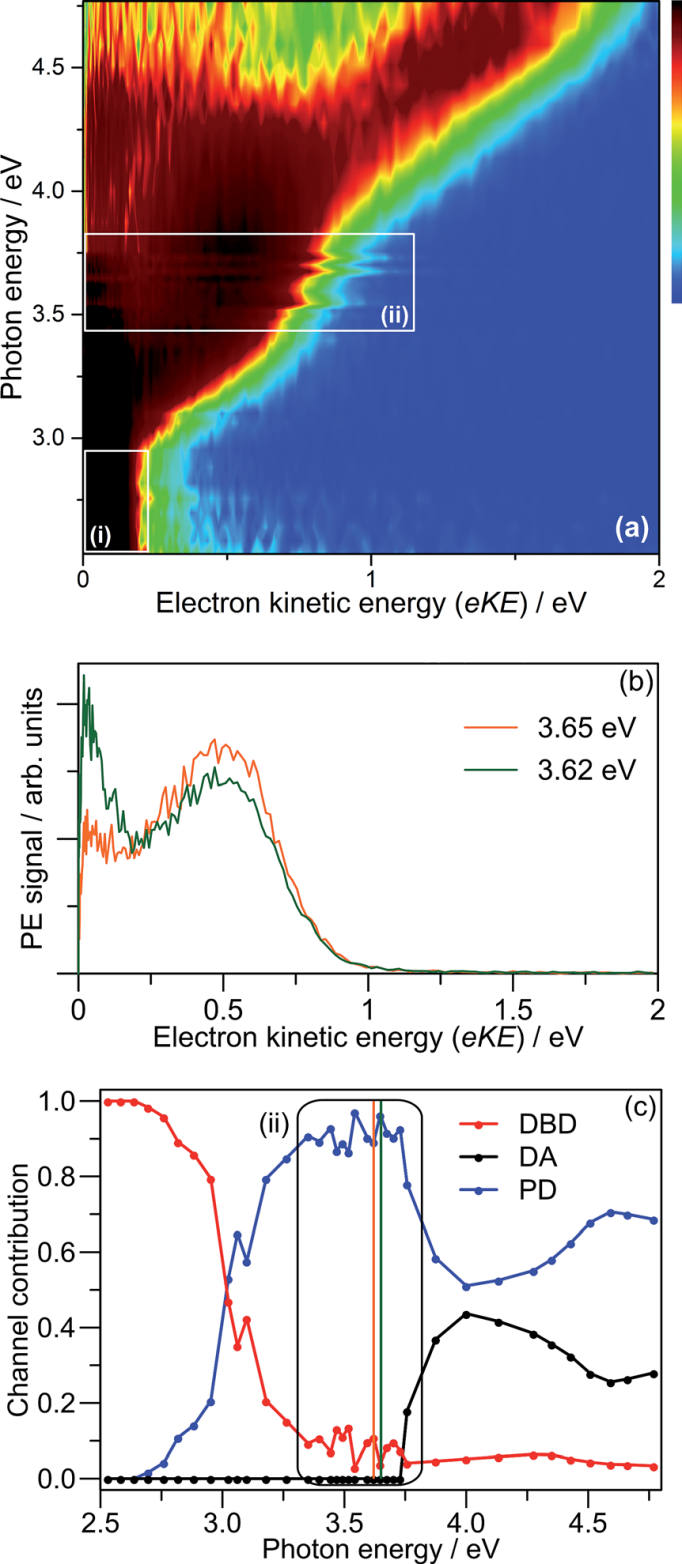
(a) Frequency-resolved photoelectron spectra of (CQ_0_)_2_^–^; (b) two example spectra from region (ii); and (c) global fit contributions of each detachment channel from (a). The orange and green bars in (c) indicate the two spectra in (b).

Fig. 3(a) shows the frequency-resolved PE spectra (37 in total) as a two-dimensional intensity plot. Each spectrum has been normalised to have unit total area for clarity. A number of trends are immediately evident. For *hν* < 3.0 eV, denoted as region (i), all PE spectra are essentially identical (see Fig. 2(c)). Between 3.3 ≥ *hν* ≥ 3.7 eV, there are two PE features: a high-eKE feature that is broadly consistent with prompt detachment; and the low-eKE feature observed for *hν* < 3.0 eV. Region (ii) in Fig. 3(a) shows a modulation in intensity between these high-eKE and low-eKE features. Two representative PE spectra from region (ii) are shown in Fig. 3(b), which illustrate that depletion of high-eKE signal is concomitant with an increase of low-eKE signal. Between 4.0 ≥ *hν* ≥ 4.5 eV the PE spectra resemble that of the isolated monomer (Fig. 2(a)),^28^ although (CQ_0_)_2_^–^ has an increased yield of PE signal in the eKE ≤ 0.2 eV range.

To analyse the detachment channel contributions in Fig. 3(a), all frequency-resolved PE spectra were fitted with a three channel global model that is detailed in the ESI.† The three channels are labelled as DBD, DA, and PD. The DBD channel describes the low-eKE vibrational distribution shown in Fig. 2(c). The DA feature is centred at eKE = 0.22 ± 0.04 eV regardless of *hν*, while the centre of the PD feature increases linearly with *hν*. The relative contributions of the three channels are shown in Fig. 3(c). An example fit of each channel is given in the in *hν* = 4.66 eV PE spectrum in Fig. 2(a). Fig. 3(c) shows that the DBD channel is dominant for *hν* < 3.0 eV, while the PD channel becomes dominant for *hν* > 3.0 eV. The modulation in region (ii) is reproduced between the PD and DBD channels. For *hν* > 3.5 eV, the DA channel becomes available and the contribution of the DBD channel is minimal (∼5% at *hν* = 4.66 eV).

The adiabatic detachment energy (ADE) was determined in the global fit by extrapolating the rising edge of the PD feature for all frequency resolved PE spectra. Similarly, the vertical detachment energy (VDE) was determined from the maximum of the PD feature in the global fit. These data are tabulated in Table 1. Note that some PE signal can be observed below ADE due to the finite temperature of the ions (∼300 K), which corresponds to ∼0.6 eV of internal energy.

**Table 1:** (CQ_0_)_2_^–^ ground state energetics in units of eV^*a*^

Method	BDE	VDE	ADE
ωB97XD//GEN1	0.93 (0.81)	2.84 (2.85)	2.51 (2.50)
MP2//GEN1^*b*^	1.37 (0.82)	2.45 (2.45)	—
ωB97XD/GEN2^*c*^	0.85 (0.81)	2.83 (2.83)	2.42 (2.42)
Experimental	1.0 ± 0.2	2.87 ± 0.03	2.6 ± 0.1

^*a*^Key: BDE – adiabatic bond dissociation energy; VDE – vertical detachment energy; and ADE – adiabatic detachment energy. Counterpoise-corrected values are in parentheses.

^*b*^This method provides a reasonable treatment of dispersion interactions (ref. 62).

^*c*^ωB97XD//GEN1 geometry. All calculated values include zero-point energy corrections.

PE angular distributions associated with Fig. 3(a), quantified in terms of the conventional *β*_2_ parameter (–1 ≤ *β*_2_ ≤ 2),^55^,^56^ are given in the ESI.† *β*_2_ values of –1 and +2 correspond to electron ejection perpendicular and parallel to the laser polarisation, ***ε***, respectively. In contrast to CQ_0_^–^,^28^ the angular distributions do not exhibit changes in anisotropy that could reflect changes in detachment channel contributions or dynamics.

### Photodetachment yield spectroscopy

**Fig. 4:**
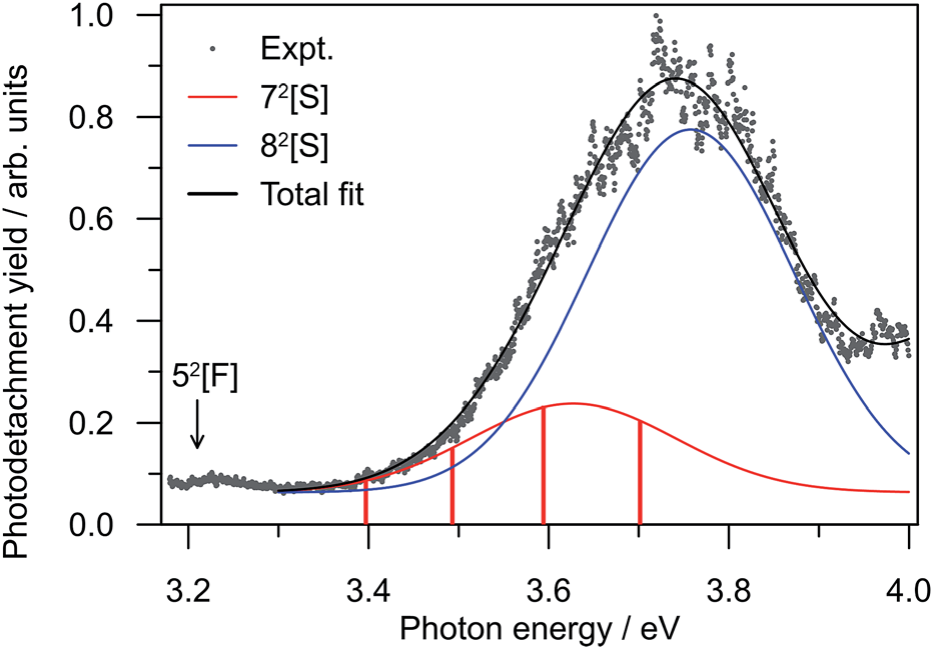
Relative photodetachment yield (action spectrum) of (CQ_0_)_2_^–^ in the 3.20 ≥ *hν* ≥ 3.88 eV window, which spans region (ii) in Fig. 3. The red bars correspond to the photon energies in region (ii) in Fig. 3 at which the DBD channel is enhanced.

To further investigate the DBD and PD channel modulation in region (ii) (Fig. 3(a)), the total photodetachment yield spectrum spanning 3.2 < *hν* < 4.0 is given in Fig. 4. The yield spectrum shows a broad maximum centred at *hν* ∼ 3.75 eV, with its red edge overlapping with the modulations. While the total PE yield shows some reproducible closely-spaced oscillations, their extent does not account for the observed channel modulation. Instead, the modulations appear to result from a competition between processes yielding the two detachment channels rather than any sharp changes in the total photodetachment cross-section. Hence, the presentation of normalised PE spectra in Fig. 3(b) is representative of their relative intensity.

### Ground state calculations

The calculated minimum energy geometry of (CQ_0_)_2_^–^ is shown in Fig. 1(b). The structure exhibits a distorted π-stack involving five hydrogen bonds of length < 2.8 Å. The separation between the centres of each *para*-quinone ring is 3.9 Å, which is the same as the neutral π-stacked benzene dimer.^57^ The two monomers in the (CQ_0_)_2_^–^ equilibrium geometry (Fig. 1(b)) are not geometrically equivalent. The left monomer has an almost planar *para*-quinone ring with carbonyl (C=O) bond lengths of ∼1.24 Å. The right monomer has a non-planar geometry, in which the C=O groups are bent out of the ring plane by ∼12°, and the C=O bond lengths are ∼1.20 Å. In contrast, both monomers are planar with all C=O bond lengths of ∼1.20 Å in the optimised (CQ_0_)_2_ geometry.

The extent to which the anionic ground state is localised or delocalised can be estimated from population analysis of each monomer. The MP2//GEN1 calculations produced MP2-density Mulliken (natural bond order, NBO,^58^ in parentheses) charges of –0.94 (–0.96) and –0.06 (–0.03) for the planar and non-planar monomers, respectively, implying a ground state anion in which the electron is localised on the right hand (non-planar) monomer in Fig. 1(b). The ωB97XD/GEN2 and CASSCF calculations gave similar populations. The non-planar geometry of one of the monomers in the dimer anion but not in the dimer neutral appears to result from differing dispersion interactions between the two species.

Systematic conformation searches (from both semi-empirical UPM6 geometries and hand-oriented starting geometries that were re-optimised at the ωB97XD//GEN1 level of theory) support the structure shown in Fig. 1(b) should be statistically predominant (>95%) in the experiment, assuming electrospray and ion thermalisation (trapping) recovers thermodynamic structures.^49^

Calculated photodetachment energetics (including zero-point energy) are summarised in Table 1, and are overall in very good agreement with experiment. The five hydrogen bonds identified in Fig. 1(b) are the main dimer cohesion force.^59^ The calculated adiabatic bond dissociation energy (BDE) of ∼0.8 eV is in reasonable agreement with that determined from the experiment (1.0 ± 0.2 eV) using the approximate relation: ADE[(CQ_0_)_2_^–^] ≈ BDE[(CQ_0_)_2_^–^] + ADE[CQ_0_^–^], where ADE is the adiabatic detachment energy and ADE[CQ_0_^–^] = 1.60 ± 0.06 eV.^28^

### Excited state calculations

**Fig. 5:**
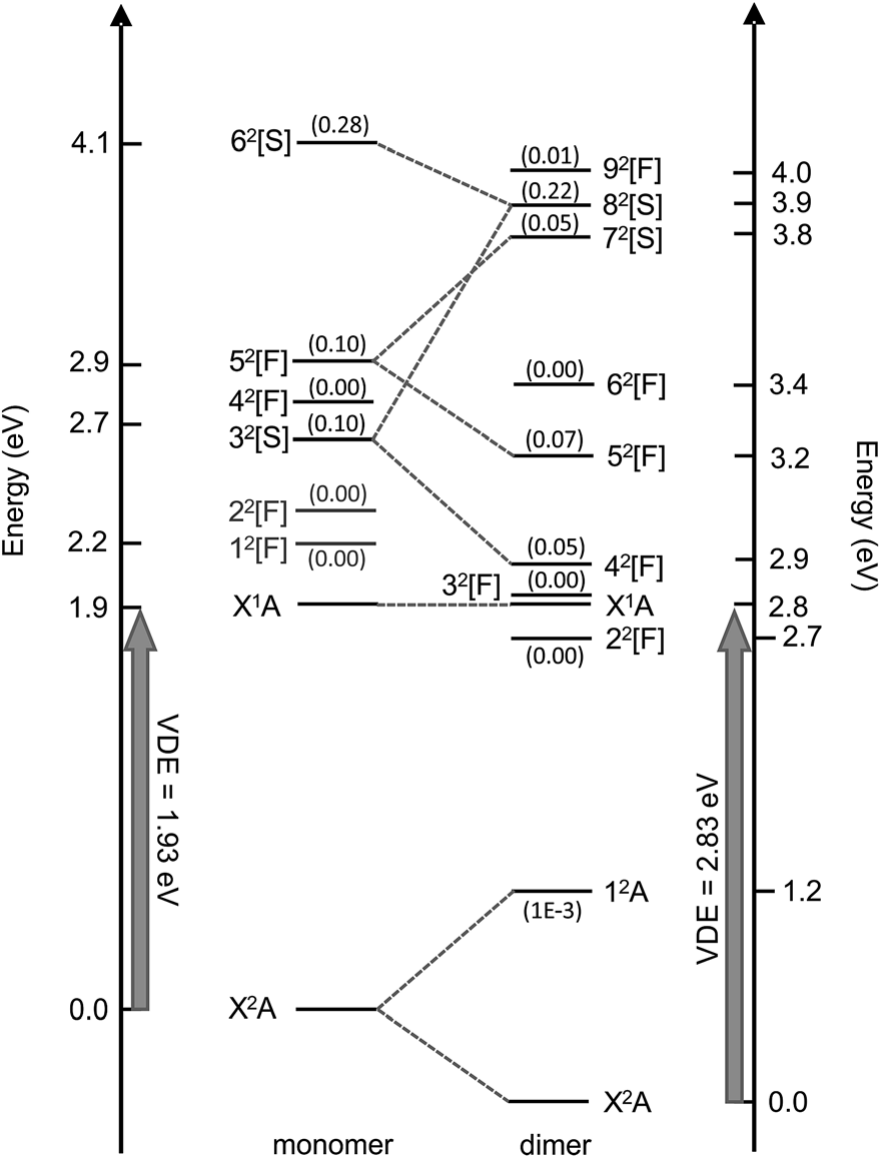
Calculated vertical electronic states and π*-resonances of (CQ_0_)_2_^–^ compared with CQ_0_^–^ (left, ref. 28). Oscillator strengths are included in parentheses. The numerical scale indicates vertical electronic energy relative to the ground electronic state (X^2^A) anion. The DBS is situated ∼150 meV below X^1^A.

Valence-localised vertical excited states of (CQ_0_)_2_^–^ are summarised in Fig. 5, and compared with similar calculations on CQ_0_^–^.^28^ The theoretical methodology, multi-state XMCQDPT2 with a large CASSCF reference active space (high static electron correlation),^53^ has been shown to perform well in a number of earlier studies.^28^,^41^,^42^,^60^ Generally, the calculation of resonances needs to be approached with particular caution in order to disentangle continuum effects.^46^,^61^ Each resonance has been categorised as having predominantly shape, [S], or Feshbach, [F], character, although most have a strongly mixed character. Full excited state wavefunction characters are summarised in the ESI.† There are five optically accessible π*-resonances in the experimental *hν* range. The 4^2^[F] and 8^2^[S] resonances predominantly involve intramolecular excitation processes on the planar monomer, while the 5^2^[F] and 7^2^[S] have a significant intermolecular or charge-transfer character. The 1^2^A excited state is a bound charge-resonance state, corresponding to the radical anion localised almost exclusively on the non-planar monomer.^21^,^22^

The first excited neutral state is vertically situated at *hν* ∼ 4.5 eV, while the lowest lying neutral triplet excited state is vertically situated at *hν* ∼ 5.3 eV. The dimer resonances correlating with CQ_0_^–^ 1^2^[F] and 2^2^[F] (shown in grey) have been excluded because they are of π* ← O(p) character, are optically inactive, and played no clear role in the dynamics of CQ_0_^–^.^28^,^41^,^42^

The resonance energetics and oscillator strengths allow the broad feature in the photodetachment yield spectrum (Fig. 4) to be assigned: there are two overlapping photoexcitation profiles arising from the intermolecular 7^2^[S] (oscillator strength ∼ 0.05) and intramolecular 8^2^[S] (oscillator strength ∼ 0.22) resonances. The spectrum has therefore been modelled using two Gaussians, plus an underlying baseline for prompt detachment and 9^2^[F] contributions at high *hν*.^63^ The fitted Gaussian positions are in good accord with the calculated energetics, and fitted widths of ∼0.2 eV (∼40 fs) in good accord with other studies.^20^–^28^,^40^–^42^,^49^

From the CASSCF wavefunction characters (see ESI†), Franck–Condon (FC) photoexcitation of the intermolecular 5^2^[F] or 7^2^[S] resonances involves high excitation of carbonyl wagging, ring puckering, C=O and C=C stretching modes, which are those required to achieve approximate 5^2^[F]/3^2^[F] and 7^2^[S]/5^2^[F] conical intersection geometries (see ESI†). In contrast, FC photoexcitation of the intramolecular 8^2^[S] resonances predominantly involve C=C and C=O stretching modes localised on the parent planar monomer.

### Dipole-bound state calculations

**Fig. 6:**
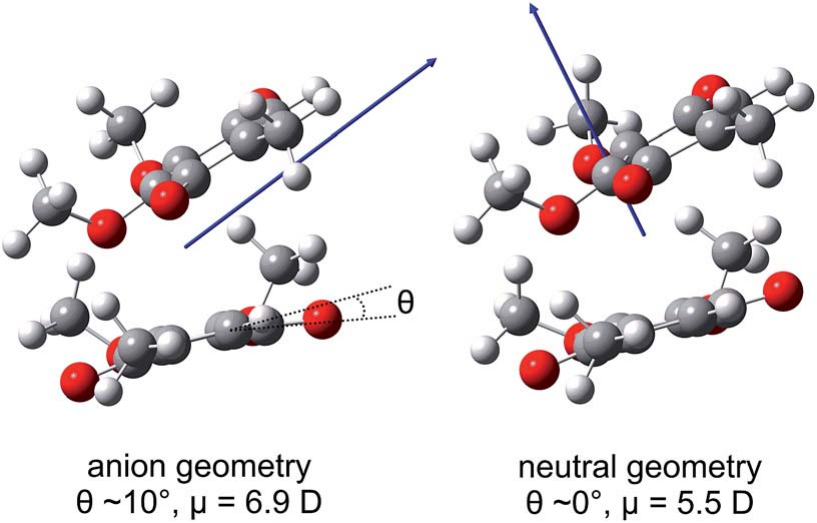
Illustrations of the ground electronic state (CQ_0_)_2_ dipole moment, *μ*, at the optimised (CQ_0_)_2_^–^ and (CQ_0_)_2_ geometries.

Calculated values of |***μ***| in the ground electronic state of (CQ_0_)_2_ are ∼6.9 D and ∼5.5 D at the optimised (CQ_0_)_2_^–^ and (CQ_0_)_2_ geometries, respectively, supporting DBSs with binding energy of ∼150 meV and ∼50 meV. However, the oscillator strength for direct photoexcitation of the DBS is ∼10^–4^ to 10^–5^, which is small compared with those for valence-localised resonances. Fig. 6 shows ***μ*** at the (CQ_0_)_2_^–^ and (CQ_0_)_2_ geometries. At the (CQ_0_)_2_^–^ geometry, ***μ*** is oriented between the π-stacked monomers and close to parallel with the planar monomer ring and the chord joining the two carbonyl groups on the non-planar monomer. In contrast, at the (CQ_0_)_2_ geometry the orientation of ***μ*** is almost orthogonal to the monomer ring planes. Calculations connecting the ground electronic state (CQ_0_)_2_^–^ to neutral geometries reveal that changes of the carbonyl tilt angle, *θ*, associated with wagging modes of the non-planar monomer (and the concomitant contraction of C=O bonds on the planar monomer), are the principal geometrical changes responsible for the large change in orientation of ***μ***. These C=O wagging modes are also FC active following intermolecular photoexcitation.

The first excited (CQ_0_)_2_ state, 1^1^A at *hν* ∼ 4.5 eV (not shown in Fig. 5), has |***μ***| ∼ 7.1 D, which also supports a DBS with binding energy ∼ 180 meV.

### Time-resolved photoelectron imaging

The dynamics and origin of the DBD channel were investigated in real-time using pump–probe femtosecond PE imaging following photoexcitation of the charge-transfer 5^2^[F] resonance. In these measurements, a femtosecond pump pulse photoexcites at *hν* ∼ 3.10 eV, and a second probe pulse monitors the excited state population after some time delay, Δ*t*.

**Fig. 7:**
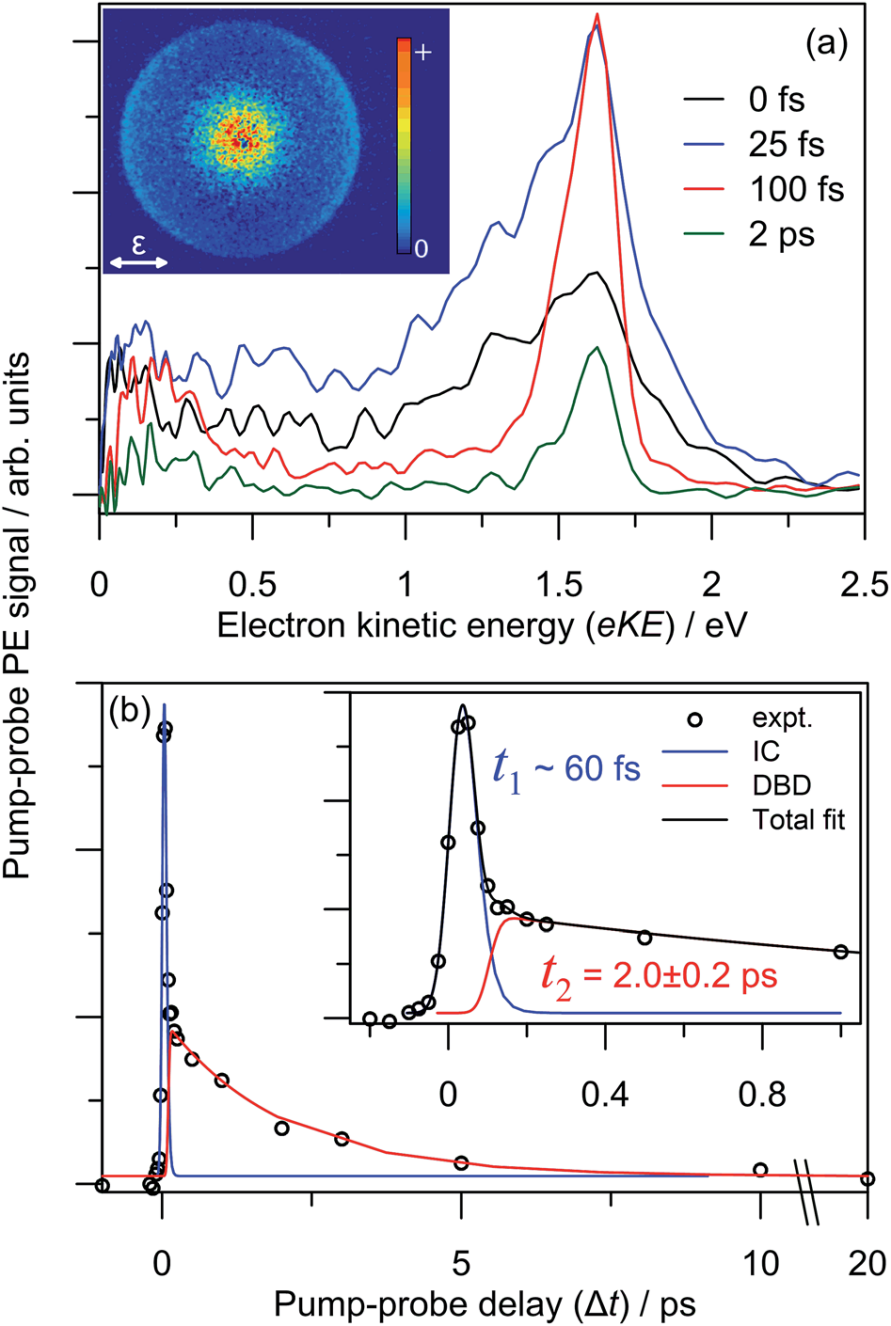
3.10 + 1.55 eV time-resolved dynamics: (a) four example Δ*t* back-ground-corrected pump–probe spectra; (b) pump–probe signal (zoomed region in inset) with fitted internal conversion (IC, blue) and dipole-bound anion detachment (DBD, red) lifetimes. The 100 fs pump–probe velocity-map image is inset in (a), illustrating *β*_2_ ∼ +2 (relative to the polarisation, *ε*) for the high-eKE feature.

Results of the 3.10 + 1.55 eV (pump + probe) time-resolved measurements are summarised in Fig. 7. Fig. 7(a) shows four selected pump–probe spectra, obtained by subtracting the background spectra (for Δ*t* ≪ 0) from each of Δ*t* ≥ 0 spectra. Fig. 7(b) shows the total integrated pump–probe PE signal as a function of Δ*t*, which reveals two timescales. The total PE signal was fitted with two functions: a Gaussian cross-correlation convoluted with an exponential decay for the fast component; and a cross-correlation function convoluted with an exponential rise and decay for the slow component. The fast component lifetime, *t*_1_ ∼ 60 fs, is limited by the experimental cross-correlation. The slow component reaches a maximum contribution after the fast component has decayed, and subsequently decays with a lifetime of *t*_2_ = 2.0 ± 0.2 ps. From Fig. 7(a), *t*_1_ is associated with the development and evolution of a broad PE feature into two narrow features, peaking at low-eKE and eKE = 1.6 eV. The high eKE feature is very close to the 1.55 eV probe energy and has a highly anisotropic PE angular distribution with *β*_2_ ∼ +2 (see inset). The anisotropy suggests that the outgoing photoelectron has p-wave character, and therefore that the original orbital from which the electron is detached has s-character.^55^ Timescale *t*_2_ is associated with the concerted decay of both pump-probe features.

**Fig. 8:**
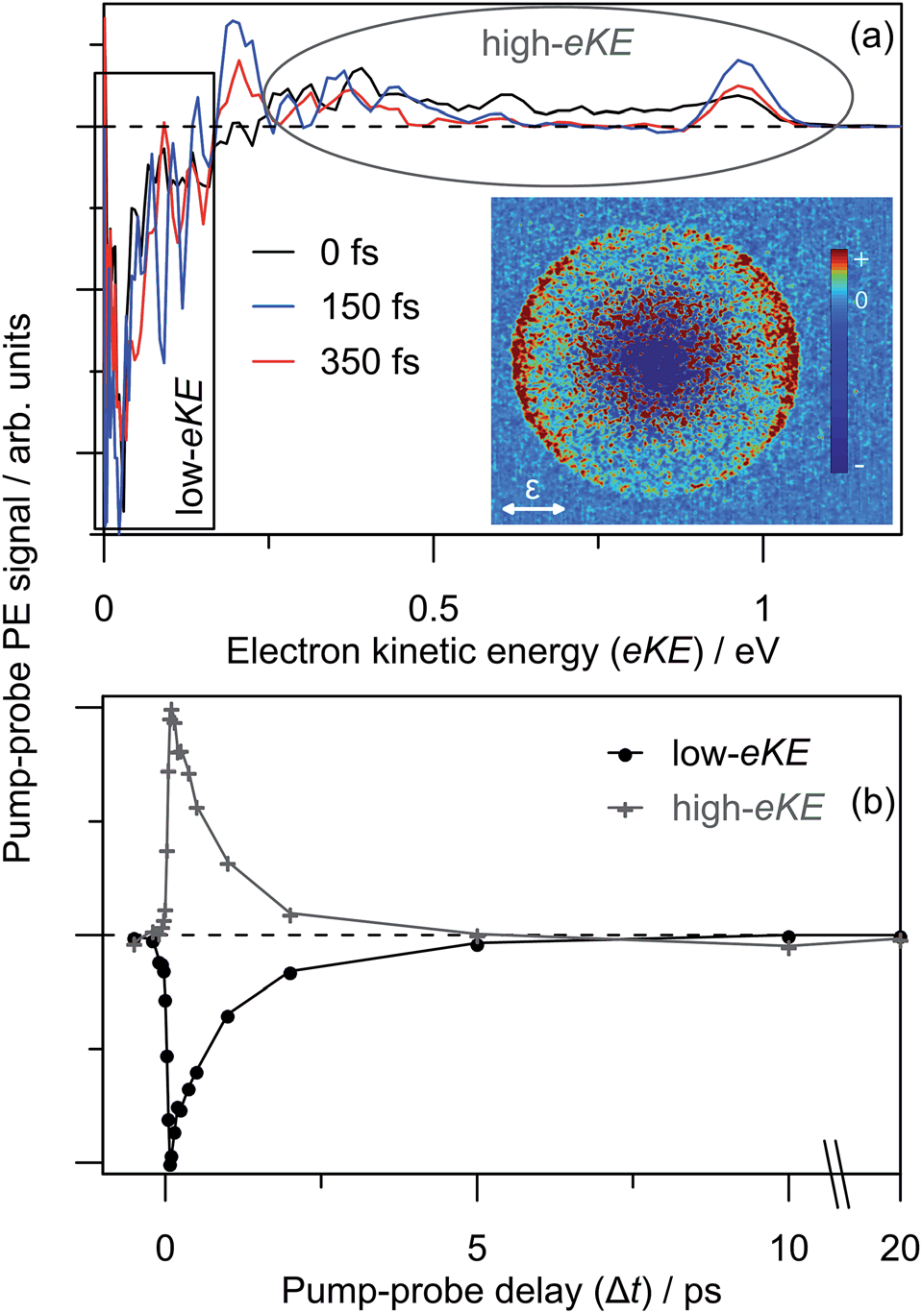
3.10 + 1.05 eV time-resolved dynamics: (a) three example Δ*t* spectra, which are divided a low-eKE and high-eKE contributions; and (b) integrated contributions of each the two pump–probe contributions. The 150 fs pump–probe velocity-map image is inset in (a), illustrating *β*_2_ ∼ +2 (relative to the polarisation, *ε*) for the high-eKE feature.

Similar time-resolved measurements were performed with a 1.05 eV probe, which are summarised in Fig. 8. Selected background-subtracted pump–probe spectra in Fig. 8(a) show an initial broad distribution in the eKE > 0.25 eV window that rapidly sharpens (sub-100 fs, *t*_1_) to a narrow distribution. This PE feature is again situated at the probe photon energy with *β*_2_ ∼ +2 angular character, and decays on a *t*_2_ ∼ 2.0 ps timescale. These observations are in agreement with the 1.55 eV probe experiments. However, concerted with the changes at high-eKE, there is now a bleach of the eKE < 0.25 eV signal, which recovers on the same *t*_2_ ∼ 2.0 ps timescale. Fig. 8(b) shows the integrated PE yield of the two features, which confirms that decay of signal in the high-eKE window is mirrored by recovery at low-eKE. The total time-resolved PE signal is invariant with Δ*t*.

## Discussion

### Ground electronic state (CQ_0_)_2_^–^

Our calculations show that the dimer radical anion can be thought of as a molecular cluster of type (CQ_0_^–^)CQ_0_, composed of a localised planar monomer anion solvated by the second non-planar monomer. The frequency-resolved PE spectra broadly support this conclusion based on the similarity with CQ_0_^–^ spectra when *hν* is red-shifted by ∼1.2 eV (Fig. 2). At high *hν*, the eKE distribution that increases commensurate with *hν* can be assigned to prompt detachment, PD, which arises from the combination of direct photodetachment into the continuum and fast autodetachment from resonances without significant nuclear motion.

### Dipole-bound state: identification, formation and autodetachment

The DBD channel in Fig. 2 and 3 is characterised by partially resolved vibrational structure at low-eKE (Fig. 2(c)). The observation of discrete vibrations combined with an eKE distribution that does not change with *hν* indicates an indirect vibration-mediated detachment. Since no similar detachment channel was observed with CQ_0_^–^,^28^ the channel must result from dimer formation. The time-resolved measurements provide an unequivocal assignment of the DBD channel to result from autodetachment of a DBS. That is, the sharp pump–probe feature suggests that the potential energy surfaces for the excited anion state and the neutral ground state are similar, while the close correlation between the probe energy and the eKE of the peak suggest a very weakly bound excited state. In addition, the PE angular distribution implies that the orbital from which the electron is detached has s-character, also supporting a DBS. The associated depletion of the DBD feature in Fig. 8(b) arises because population is removed from the DBS by the probe. However, the bleach of the DBD channel is not observed with the 1.55 eV probe, probably because this probe is close to the energy difference between the neutral X^1^A and 1^1^A states. Since both of these neutral states support a DBS, photoexcitation between the two DBS is probably efficient due to a large photoexcitation cross-section. Autodetachment from the DBS associated with the 1^1^A neutral will lead to low-eKE signal that overlaps with the X^1^A state DBD depletion.

Before the DBS is formed, the time-resolved measurements showed a transient broad PE feature that sharpens to the DBS feature on a *t*_1_ < 60 fs lifetime. At the initial pump energy of 3.10 eV, photoexcitation is resonant with the optically-active 5^2^[F] resonance, which in accord with our other studies on monomer *para*-quinone anions,^28^,^40^–^42^ should produce a broad pump–probe feature. The evolution of this broad PE feature into the narrow DBS feature reflects internal conversion of 5^2^[F] population to the DBS on a <60 fs timescale. The internal conversion lifetime of <60 fs is similar to those measured in our earlier *para*-quinone monomer anion studies.^28^,^40^–^42^ From our calculations, we postulate that 5^2^[F] population first undergoes internal conversion to the 3^2^[F] resonance, which then converts to the DBS. The 3^2^[F] resonance is predominantly localised on the non-planar monomer and energetically situated close to threshold. A calculated 3^2^[F]/5^2^[F] conical intersection geometry, given in the ESI,† indicates motion along the expected FC modes to support a fast and efficient internal conversion. Following the 5^2^[F] → 3^2^[F] internal conversion, the original 5^2^[F] FC motion on the 3^2^[F] surface directs the geometry of the non-planar monomer towards that of CQ_0_^–^. Concerted with the geometrical changes of the non-planar monomer is a decrease of the energy of the 3^2^[F] resonance to become a valence-bound state situated 0.1–0.2 eV below threshold (analogous to 2^2^[F] in Fig. 5). 3^2^[F] is similarly situated below the detachment threshold at the (CQ_0_)_2_ optimised geometry. Thus, ideal conditions for internal conversion to a DBS are achieved: the original FC photoexcitation modes of 5^2^[F] modulates the 3^2^[F] state over the DBS, which facilitates a curve crossing. The subsequent *t*_2_ = 2.0 ± 0.2 ps lifetime of the DBS can be assigned to its autodetachment lifetime and leads to the vibrational structure observed in the DBD channel as seen in Fig. 2(c).

**Fig. 9:**
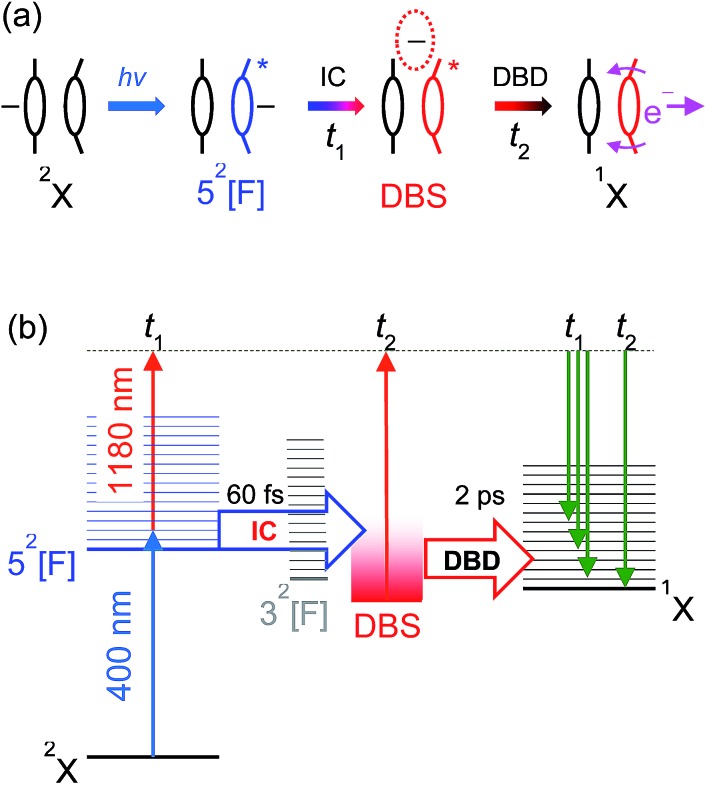
(a) Summary of the 5^2^[F] photoexcitation dynamics; (b) schematic summary of the 3.10 + 1.05 eV experiments. Key: IC (*t*_1_) – internal conversion; DBD (*t*_2_) – dipole-bound detachment.

The overall time-resolved dynamics are summarised schematically in Fig. 9(a), while the detailed interpretation of the 3.10 + 1.05 eV time-resolved measurements are summarised in Fig. 9(b). Note that the *t*_1_ timescale corresponds to only a few vibrational periods, implying that extensive intramolecular vibrational relaxation away from FC modes is unlikely.

The vibrational structure associated with the DBD channel in the frequency-resolved spectra (Fig. 2(c)) indicates that the DBS autodetachment is mode-specific. The most likely vibrational modes responsible are the original FC-active carbonyl wagging modes; these modes induce a large change in orientation of ***μ*** (see Fig. 6), which is a primary condition that influences the lifetime of a DBS.^64^,^65^ That is, the carbonyl wagging motion strongly modulates the orbital defining the DBS, which induces a coupling between the neutral and DBS potential energy surfaces. The result is the wagging motion shakes off the weakly bound electron at a kinetic energy proportional to the wagging frequency in accord with the propensity rule for the vibrational quantum number to be reduced by one.^65^,^66^ The calculated energies of the three relevant wagging modes (see ESI†) have been included in Fig. 2(c), which agree well with the DBD vibrational structure. The measured DBS lifetime of *t*_2_ = 2.0 ± 0.2 ps represents the average vibration-mediated autodetachment rate from all contributing vibrational modes. Taking the three groups of wagging modes shown in Fig. 2(c) to have wavenumbers of 200 cm^–1^, 400 cm^–1^, and 800 cm^–1^, the DBS electron is shaken off over ten to forty vibrational wags. In principle, each mode may be expected to exhibit a unique lifetime, which may be observable by integrating the recovery of the DBD feature over specific vibrational modes. However, our data is unable to resolve such a situation for (CQ_0_)_2_^–^.

Time-resolved dynamics involving DBSs have been implicated by the Neumark group in their femtosecond PE spectroscopy experiments following photoexcitation of iodine anions coordinated to CH_3_CN, CH_3_NO_2_, or nucleobases.^67^–^70^ CH_3_CN (|***μ***| ∼ 3.9 D), which does not support a valence-bound anion, exhibits DBS autodetachment on a 4–900 ps timescale.^67^ In contrast, the DBS of CH_3_NO_2_ (|***μ***| ∼ 3.5 D) converts on a ∼400 fs timescale to a valence-bound anion situated ∼100 meV lower in energy, which is facilitated by a similar vibrational wagging and modulation of ***μ*** to that for (CQ_0_)_2_^–^.^71^–^73^ Similar studies on nucleobases characterised DBS lifetimes of 0.3–11 ps before internal conversion to a valence-bound anion situated close in binding energy to the DBS.^68^–^70^ Again, electronic structure calculations suggest internal conversion to be facilitated through ring puckering and wagging modes.^68^–^70^,^74^–^76^ The dynamics characterised in the present study suggest that a valence excited state (the 3^2^[F] or 5^2^[F] resonance) can also evolve into a DBS. However, in contrast to the Neumark studies, (CQ_0_)_2_^–^ does not undergo internal conversion from the DBS to the lower-lying 1^2^A and X^2^A states, probably because any coupling would require very large geometrical distortions (that may not support a DBS), and will be unlikely on the ∼2 ps DBS autodetachment lifetime. It can therefore be concluded that internal conversion between a valance-localised state and a DBS, in either direction, requires near degeneracy. These trends provide further confirmation that the 3^2^[F] resonance is likely involved in formation of the DBS rather than direct internal conversion from the photoexcited 5^2^[F] resonance.

One of the key outcomes from (CQ_0_)_2_^–^ is evidence of the extent to which the non-adiabatic dynamics are altered by π-stacking. Specifically, although the electronic ground state of the dimer represents a localised monomer that is merely solvated, a range of new non-adiabatic dynamics in the continuum are accessed due to the availability of charge-transfer excitations and a cluster DBS. This situation is likely to be common to other cluster anions with similar chromophore/electrophore groups, and means that the general extrapolation of monomer to cluster dynamics is not trivial. Nevertheless, as will be described next, monomer-localised dynamics can be observed in some circumstances.

### Competition between local and non-local dynamics in the continuum

The modulation between the DBD and PD channels in region (ii) of Fig. 3(a), which was specifically illustrated in Fig. 3(b), is now considered. From the photodetachment yield spectrum in Fig. 4, the modulation coincides with the photoexcitation of the 7^2^[S] and 8^2^[S] resonances, which have overlapping photoexcitation profiles. The 7^2^[S] resonance has orbital contributions that are localised on the non-planar monomer, while the 8^2^[S] state is almost exclusively localised on the planar monomer. The observation of a modulation usually reflects a vibrational-specific process. From the example spectra in Fig. 3(b), if the photoexcitation cross-section to the 8^2^[S] resonance is assumed to be constant (*i.e.*, prompt detachment contributions equal), there should be photoexcitation features in Fig. 4 with a ∼20% enhancement in relative intensity that are concomitant with the modulations in Fig. 3(a). However, while the photodetachment yield spectrum exhibits some sharp features, they do not correlate with the observed modulation, nor do they show a ∼20% signal enhancement. The modulation between DBD and PD channels therefore appears to arise from a competition in non-adiabatic decay channels from the 7^2^[S] and 8^2^[S] resonances. This conclusion is supported by three additional observations. First, Fig. 3(c) and 4 show that for *hν* > 3.8 eV the DBD channel yield diminishes, even though 8^2^[S] is still strongly photoexcited. Second, from Fig. 4, the modulation is situated over the fitted 7^2^[S] photoexcitation profile. Third, for *hν* > 4.0 eV, the PE spectra of (CQ_0_)_2_^–^ have a similar appearance to those for CQ_0_^–^,^28^ although blue-shifted by ∼1.2 eV.

Our calculations suggest that photoexcitation of the 7^2^[S] resonance at *hν* ∼ 3.6 eV involves similar intermolecular FC modes as the 5^2^[F] resonance. In contrast, photoexcitation of the 8^2^[S] resonance almost exclusively involves intramolecular population of π*CC orbitals localised on the planar monomer. A conical intersection, detailed in the ESI,† suggests that the 7^2^[S] and 5^2^[F] resonances are non-adiabatically connected along the FC modes in a similar way to the 5^2^[F] and 3^2^[F] resonances. Thus, a fast internal conversion of 7^2^[S] population to 5^2^[F] could be expected. In turn, the 5^2^[F] resonance can form the DBS as above. Interestingly, the observed modulations in Fig. 3(a) and (c) have a spacing of ∼0.1 eV, which is similar to the wavenumber of the main intermolecular FC wagging modes (and IR active) calculated at ∼800 cm^–1^.

For 3.55 < *hν* < 3.8 eV, the 8^2^[S] resonance is predominantly photoexcited since it has a much higher oscillator strength. As this resonance is predominantly localised on the planar monomer, photoexcitation will involve intramolecular FC modes. From Fig. 3(a) and (c), 8^2^[S] photoexcitation does not result in efficient internal conversion to the DBS, rather the PD channel dominates. For *hν* > 3.75 eV, in which 8^2^[S] is exclusively photoexcited, the competition between DBD and PD channels is no longer observed. Instead, the DA feature centred at eKE = 0.22 ± 0.04 eV becomes available. The DA feature energetically correlates with a delayed autodetachment from the 4^2^[S] resonance, which is also predominantly localised on the planar monomer. The assignment of DA to the 4^2^[S] resonance suggests an internal conversion route from 8^2^[S] photoexcitation. Because extensive nuclear motion (or energy redistribution) cannot occur on the ultrafast lifetimes of resonances, the internal conversion will be facilitated through intramolecular FC modes. In accord, the non-planar monomer effectively acts as a spectator so the detachment dynamics are monomer-like in character.^28^ This conclusion is also supported from the *hν* > 3.75 eV PE spectra, which have a similar appearance to those of CQ_0_^–^,^28^ except blue shifted by ∼1.2 eV due of the increased electron affinity of the dimer.

In summary, the modulation between the DBD and PD channels in the 3.4 ≥ *hν* ≥ 3.8 eV range likely results from a mode-specific competition between non-adiabatic decay pathways of the 7^2^[S] and 8^2^[S] resonances. Such dynamics can only be uncovered by frequency-resolved PE spectroscopy combined with relative cross-section (total PE yield) measurements. A detailed theoretical account of such dynamics will likely be very challenging, particularly due to the participation of the continuum. However, despite the interplay and competition of multiple channels, it is remarkable and encouraging that the technique of frequency-, angle-, time-resolved imaging can provide such rich insight. Overall, to the best of our knowledge, this study presents the first characterisation of a non-adiabatic dynamics competition between resonances with varying inter- and intramolecular character.

## Conclusions

We have demonstrated that intermolecular or charge-transfer photoexcitation can play a significant role in the excited state non-adiabatic dynamics of a π-stacked dimer radical anion. We have provided the first direct and real-time evidence of internal conversion of above-threshold resonances into a cluster-supported DBS, and its subsequent vibration-mediated autodetachment. Formation of the DBS is facilitated through charge-transfer photoexcitation by virtue of π-stacking. However, despite the additional complexity introduced by dimerization, monomer-like dynamics can also be observed following photoexcitation of resonances primarily localised on the monomer that supports the excess electron in the dimer ground electronic state. When both inter- and intra-molecular resonance photoexcitation profiles overlap, a remarkable competition between ‘dimer’- and ‘monomer’-like non-adiabatic dynamics has been observed. Such interplays between inter- and intramolecular non-adiabatic dynamics are likely to be common in other similar π-stacked cluster anions, and further illustrate the rich dynamics that can occur in the detachment continuum.

## Supplementary Material

Supplementary information
