# Supplementary material for: Ultrafast dynamics of formation and autodetachment of a dipole-bound state in an open-shell π-stacked dimer anion
Source: Chem Sci. 2016 May 4;7(8):5352–61. doi: 10.1039/c6sc01062h (PMC6020752; doi:10.1039/c6sc01062h)
Supplement: Supplementary file 1 [file SC-007-C6SC01062H-s001.pdf]

Electronic Supplementary Material (ESI) for Chemical Science.
This journal is © The Royal Society of Chemistry 2016

Electronic Supplementary Information for 'Ultrafast dynamics of the formation and autodetachment of a dipole-bound state in an open-shell π-stacked dimer anion'

James N. Bull[1], Christopher W. West, Jan R. R. Verlet

Department of Chemistry, Durham University, South Road, DH1 3LE, United Kingdom. E-mail: j.r.r.verlet@durham.ac.uk
[1]Current address: School of Chemistry, University of Melbourne, Parkville, Melbourne VIC 3010, Australia. E-mail: james.bull@eigenket.org

Experimental

Experiments were performed using a photoelectron (PE) imaging spectrometer that has been previously described in detail.[1-4] Briefly, a ~2 mM solution of >99% purity CQ_0 (Sigma-Aldrich) dissolved in analytical grade methanol was electrosprayed and transferred *via* a vacuum transfer capillary into an RF ring-electrode ion trap. The abundance of CQ_0^- to $(CQ_0)_2^-$ was roughly optimized by varying the flow of compressed air over the electrospray needle, and varying the separation between the electrospray needle and transfer capillary. Ions are thermalized to ~300 K in the trap (total vibrational internal energy is ~600 meV). The trapped ions are unloaded into a co-linear time-of-flight optics assembly in which the ion packet is accelerated along a 1.3 m flight region towards a continuous-mode penetrating field velocity-mapping (VMI) assembly.[5,6] Laser pulses were timed to interact with the mass-selected ion packet at the centre of the VMI stack. Ejected electrons were velocity-mapped onto a dual chevron multichannel plate (MCP) detector followed by a P43 phosphor screen, which was monitored by a CCD camera (~512×512 pixels). The electron kinetic energy (*eKE*) scale was calibrated from the spectrum of I^-, and the velocity-mapping resolution is around 5%. All velocity-map image reconstructions used a polar onion peeling (POP)[7] algorithm providing the PE spectrum and electron ejection angular distributions.[8] Identification of the dimer species in the time-of-flight mass spectrum was achieved based on an accurate mass calibration using the deprotonated isotopomer spectrum of 1,6-dibromo-2-hydroxynaphthalene-3-carboxylic acid.

In the frequency-resolved experiments, PE velocity-map images were collected at various wavelengths between hv = 4.77 eV (260 nm) and hv = 2.53 eV (490 nm), in 5 nm or 10 nm increments. PE signal rapidly disappeared below 490 nm. The tuneable radiation was generated by a Nd:YAG (Continuum Surelite II-10) pumped optical parametric oscillator (Continuum Horizon I, ~6 cm^{-1} resolution). Each image accumulated sufficient counts to achieve a reasonable and comparable signal-to-noise ratio. All images were accumulated with a 500 ns MCP gate; it was observed by systematically stepping the gate width and delay parameters that all electron ejection occurs within 50 ns following the laser pulse (*i.e.*, no thermionic emission).[2,9-13] Below hv < 2.8 eV, PE signal rapidly decreases, indicating insufficient hv to induce any detachment process.

The relative photodetachment yield (cross-section) as a function of hv was determined as the average of ten runs, and was corrected for laser power from a similar measurement of the photodetachment yield from I^-. The relative error, determined as twice the standard deviation of all runs, is ~6%.

In the time-resolved (pump-probe) experiments, femtosecond laser pulses were derived from a Spectra Physics Ti:sapphire oscillator and regenerative amplifier. Pump and probe pulses were delayed relative to each other, Δt, using a motorized delay line. Two different pump-probe schemes were studied: (i) 3.10 eV (400 nm, ~80 μJ) pump and 1.55 eV (800 nm, ~300 μJ) probe; and (ii) 3.10 eV (400 nm, ~80 μJ) pump and 1.05 eV (1180 nm, ~100 μJ) probe. Combined, the pump-probe cross-correlations are FWHM is ~60 fs. The ultimate time resolution is approximately half the FWHM cross-correlation. The 3.10 eV photons were produced by frequency doubling of the 1.55 eV (800 nm) fundamental in a type I β-barium borate crystal (BBO). In a given time-resolved experiment, pump and probe pulses were combined collinearly using a dichroic mirror, and were loosely focused into the interaction region using a curved metal mirror.

Theoretical

All electronic structure calculations were performed using the Gaussian 09 and GAMESS-US software packages.[14,15] Possible dimer radical anion geometries were initially explored using both the UPM6 semi-empirical Hamiltonian and hand-oriented geometries using chemical sense.[16] Candidate geometrical minima were reoptimized using the

ωB97XD//GEN1 and MP2//GEN1 levels of theory.[17] The ωB97XD functional was chosen due to reasonable performance in reproducing electron affinities, hydrogen-bonding and non-covalent interactions, as well as excited states that involve considerable charge-transfer character.[17-20] The GEN1 molecular basis set is the aug-cc-pVDZ basis set excluding the most diffuse set of *d* functions on carbon atoms.[21] Similarly, the GEN2 molecular basis set is the aug-cc-pVTZ basis set excluding the most diffuse set of *f* functions on carbon and oxygen atoms, and the most diffuse set of *d* functions on carbon atoms. All geometrical minima were confirmed to represent true minima though calculation of harmonic vibrational frequencies. Basis set superposition errors were incorporated using Counterpoise (CP) method.[22]

Resonance energetics were calculated at the multistate XMCQDPT2/GEN1 level of theory with a CASSCF(9,12) reference wavefunction assuming the MP2//GEN1 or otherwise stated geometry.[23] Energetics at the CASSCF geometry were nearly identical to those at the MP2 geometry. The CASSCF reference included six π^* orbitals and all bar the four lowest energy π orbitals for computational tractability. TD-DFT calculations indicated the π and π^* orbitals excluded from the CASSCF active space to have minimal contribution to the resonances considered in this study. A complete π and π^* active space was not computationally feasible. Our earlier combined experimental and theoretical studies on π^* resonances of *para*-quinone monomer anions support that multistate XMCQDPT with a sufficiently large reference active space and sufficiently compact valence-localized basis set can provide a reasonable account of resonance vertical photoexcitation energetics.[1-3] Oscillator strengths were computed within the CAS-CI framework. Approximate conical intersection geometries were located using the NACME method with state-averaged CASSCF wavefunctions.[24]

The dipole-bound state binding energy was approximated in the TD-DFT framework at the optimized anion and neutral geometries.[25] Briefly, all atoms were treated with the 6-31+G** basis set,[26] which was augmented with six uncontracted *sp* and *d* functions, whose orbital exponents become increasingly smaller (i.e., more diffuse) following a geometrical progression (factor 3.2). The computed binding energies are in accord with correlations between dipole moments and experimental DBS binding energies.[27,28]

Results and analysis

Angle-resolved photoelectron imaging

PE angular anisotropy (β_2) from the velocity-map images of $(CQ_0)_2^-$ are summarized in Fig. S1. Note that a five-point moving-mean has been applied to all β_2 data in this Fig.. Positive and negative β_2 corresponds to electron ejection parallel and perpendicular to the laser polarization, respectively.[8,29]

Frequency-resolved global fit

The global fit of all frequency-resolved spectra involved several assumptions: (i) the VAD feature is described by the vibrational fit shown in Fig. 2c; (ii) the delayed autodetachment (DA) feature assumes a Gaussian distribution convoluted with a Wigner threshold expression near zero *eKE*;[30,31] (iii) prompt detachment (PD) is modelled with a global function that takes shape similar to that for other *para*-quinones; 1 and (iv) all spectral parameters such as the *eKE* centers and spectral widths of the DA and PD features are shared and constant across all PE spectra - with the exception that the prompt detachment (PD) distribution increases linearly in *eKE* with *hv* (*i.e.*, fixed in electron binding energy). The final fit, shown in Fig. S2, was that requiring the minimum number of basis functions that reproduces the experimental data in good accord without significant and/or systematic unassigned residual, and provides a physically-meaningful interpretation.

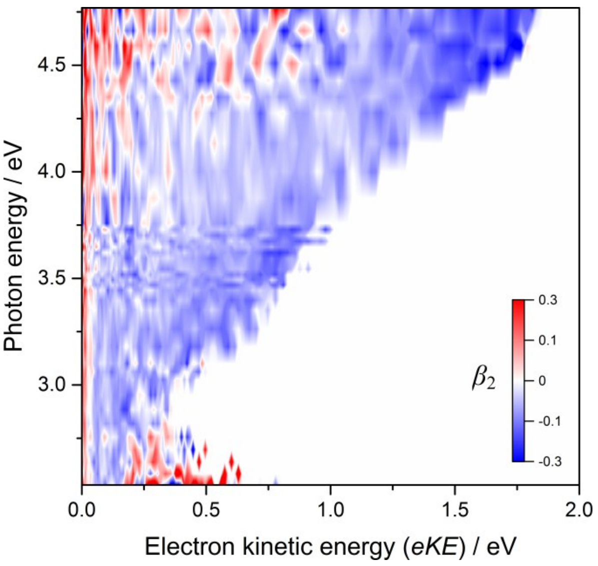

Fig. S1 PE angular anisotropy, β_2, associated with the frequency-resolved spectra of $(CQ_0)_2^-$.

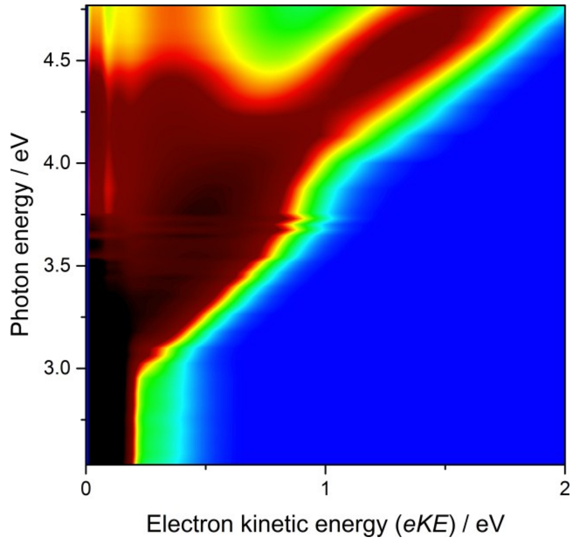

Fig. S2 Global fit of all frequency-resolved spectra.

CASSCF natural orbitals

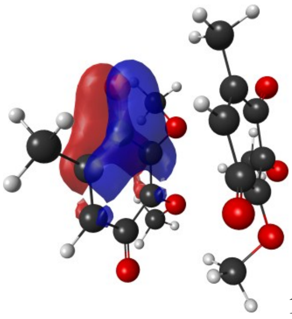

1

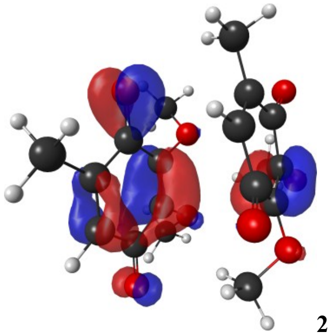

2

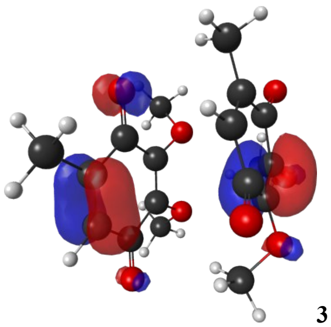

3

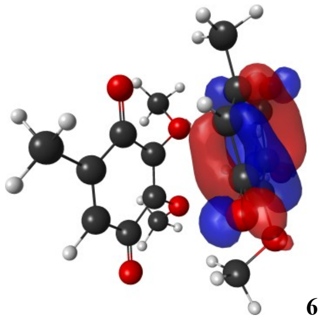

6

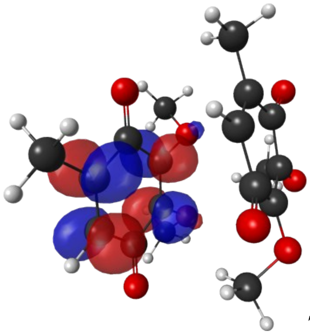

7

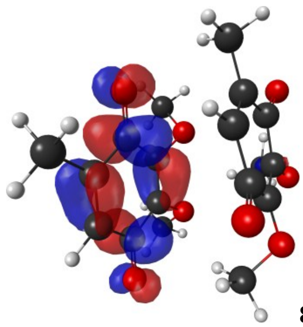

8

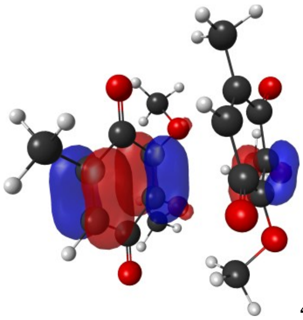

4

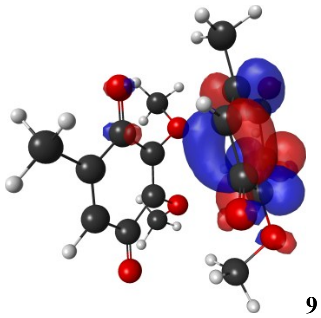

9

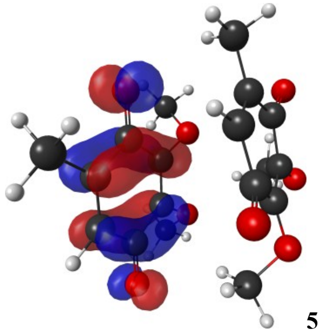

5

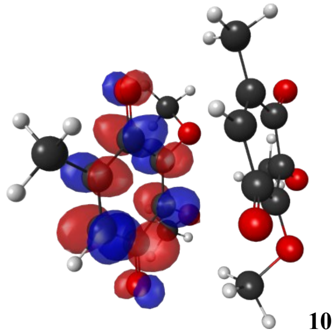

10

Predominant CASSCF wavefunction configurations (MP2//aug-cc-pVDZ $(CQ_0)_2^-$ geometry)

Key: superscript 2 = doubly occupied orbital; superscript +/- = singly occupied orbital with spin up/down; 0 = unoccupied orbtial; P = planar monomer; N = non-planar monomer; and PN = delocalized across both monomers; subscript CC and CO indicates C=C and C=O bonds, respectively. Descriptions are in order of predominant configurations. Minor configurations have been omitted.

State / resonance	Predominant configurations	Description and notes
X^2A	$1^2 2^2 3^2 4^2 5^+ 6^0 7^0 8^0 9^0 10^0$	Ground state, excess electron localized in the P(π^*_{CO}) orbital.
1^2A	$1^2 2^2 3^2 4^2 5^0 6^+ 7^0 8^0 9^0 10^0$	N(π^*_{CO}) ← P(π^*_{CO}). This is the charge-resonance state, corresponding to the ground anion state of the non-planar monomer.
2^2[F]	(38%):$1^2 2^2 3^2 4^+ 5^2 6^0 7^0 8^0 9^0 10^0$ + (27%):$1^2 2^2 3^+ 4^2 5^2 6^0 7^0 8^0 9^0 10^0$	N(π^*_{CO}) ← P(π_{CC}), P(π^*_{CO}) ← PN(π_{CC}). Both predominant configurations involve core-excitations.
3^2[F]	(29%):$1^2 2^2 3^2 4^+ 5^- 6^+ 7^0 8^0 9^0 10^0$ + (20%):$1^2 2^2 3^+ 4^2 5^- 6^+ 7^0 8^0 9^0 10^0$ + (12%):$1^2 2^2 3^2 4^+ 5^+ 6^- 7^0 8^0 9^0 10^0$	Multi-electron reconfigurations, N(π^*_{CO}) ← PN(π_{CC}). All predominant configurations involve single occupancy of the N(π^*_{CO}) orbital implying a high charge-transfer character. This state is close to threshold and able to couple to the dipole-bound state.
4^2[F]	(59%):$1^2 2^+ 3^2 4^2 5^2 6^0 7^0 8^0 9^0 10^0$ + (16%):$1^2 2^2 3^2 4^2 5^0 6^0 7^0 8^+ 9^0 10^0$	P(π^*_{CO}) ← PN(π_{CC}), P(π^*_{CO}) ← P(π^*_{CO}). This is the delayed autodetachment (DA) resonance in the frequency-resolved measurements, and is localized on the planar monomer. Resonance is optically-accessible.
5^2[F]	(27%):$1^2 2^2 3^2 4^2 5^0 6^0 7^+ 8^0 9^0 10^0$ + (27%):$1^2 2^2 3^2 4^+ 5^+ 6^- 7^0 8^0 9^0 10^0$ + (20%):$1^2 2^2 3^+ 4^2 5^+ 6^- 7^0 8^0 9^0 10^0$	P(π^*_{CC}) ← P(π^*_{CO}), N(π^*_{CO}) ← PN(π_{CC}), N(π^*_{CO}) ← PN(π_{CC}). The second configuration is in common with the 3^2[F] resonance. The latter two configurations involve single occupancy of the N(π^*_{CO}) orbital implying a moderate charge-transfer character. Resonance is optically-accessible, and is that initially photoexcited in the time-resolved measurements.
6^2[F]	(58%):$1^2 2^+ 3^2 4^2 5^+ 6^- 7^0 8^0 9^0 10^0$ + (10%):$1^2 2^2 3^+ 4^2 5^+ 6^- 7^0 8^0 9^0 10^0$	N(π^*_{CO}) ← PN(π_{CC}), N(π^*_{CO}) ← PN(π_{CC}). The last configuration is in common with the 5^2[F] resonance. All configurations involve single occupancy of the N(π^*_{CO}) orbital implying a high charge-transfer character.
7^2[S]	(53%):$1^2 2^2 3^2 4^2 5^0 6^0 7^+ 8^0 9^0 10^0$ +	P(π^*_{CC}) ← P(π^*_{CO}), N(π^*_{CO}) ← P(π_{CC}), N(π^*_{CO}) ← P(π_{CC}). The

	(17%):$1^2 2^2 3^2 4^+ 5^+ 6^- 7^0 8^0 9^0 10^0$ + (8%):$1^2 2^2 3^+ 4^2 5^+ 6^- 7^0 8^0 9^0 10^0$	latter two configurations involve single occupancy of the N(π^*_{CO}) orbital implying a moderate charge-transfer character. The first configuration is in common with the 5^2[F] resonances and the second is in common with the 5^2[F] and 3^2[S] resonances. The last configuration is in common with the 6^2[F] resonance. Overall, internal conversion with the 5^2[F] resonance is anticipated (supported in the frequency-resolved measurements). Resonance is optically-accessible.
8^2[S]	(37%):$1^2 2^2 3^2 4^2 5^0 6^0 7^0 8^+ 9^0 10^0$ + (15%):$1^2 2^2 3^2 4^+ 5^- 6^0 7^+ 8^0 9^0 10^0$ + (14%):$1^2 2^2 3^+ 4^2 5^- 6^0 7^+ 8^0 9^0 10^0$ + (12%):$1^+ 2^2 3^2 4^2 5^2 6^0 7^0 8^0 9^0 10^0$	P(π^*_{CO}) ← P(π^*_{CO}), multi-electron reconfiguration, P(π^*_{CO}) ← P(π_{CO}) + multi-electron reconfiguration. The first configuration is in common with the 4^2[F] resonance. Experimentally, internal conversion to the 4^2[F] resonance is observed following photoexcitation of this resonance. Resonance is strongly optically-accessible.
9^2[F]	(36%):$1^2 2^2 3^2 4^+ 5^- 6^0 7^+ 8^0 9^0 10^0$ + (32%):$1^2 2^2 3^+ 4^2 5^0 6^2 7^0 8^0 9^0 10^0$	Multi-electron reconfigurations with some charge-transfer character. The first configuration is in common with the 8^2[S] resonance. Resonance is optically-accessible, and may contribute to the high *hv* baseline in the photodetachment yield spectrum (Fig. 4).

Overall Shape and Feshbach character (in %) of each resonance at the MP2//aug-cc-pVDZ $(CQ_0)_2^-$ geometry

Resonance	Shape	Feshbach
2^2[F]	7	93
3^2[F]	2	98
4^2[F]	19	81
5^2[F]	29	71
6^2[F]	3	97
7^2[S]	53	47
8^2[S]	38	62
9^2[F]	3	97

Conical intersection geometries

Possible conical intersection geometries between the 5^2[F]/3^2[F], and 7^2[S]/5^2[F] resonances are summarized in the Table S1. We acknowledge that these calculations do not take into account interaction with the continuum,[32] thus these geometries should be taken as rough indications of geometrical motion leading to internal conversion. Further, such calculations are particularly difficult to compute in a dimer system due to the intermolecular co-ordinate with a shallow gradient. With these caveats, the conical intersection geometries between either intramolecular resonances or intermolecular resonances all predominantly involve changes in the carbonyl tilt angle and C=O and C=C bond lengths.

The 5^2[F]/3^2[F] conical intersection requires recoil of the non-planar monomer to adopt a geometry close to that of the planar monomer in the ground electronic state anion. In particular, the carbonyl tilt angle of the original non-planar monomer is now close to planar. These modes are those expected to be FC-active. The large stretching of one P(C=C) bond reflects that both 5^2[F] and 3^2[F] resonances involve migration of population from a P(π^*_{CC}) orbital. The 7^2[S]/5^2[F] conical intersection involves the original planar monomer becoming non-planar and *vice versa* for the other monomer, thus requiring carbonyl wagging excitation on both monomers. A conical intersection between the 8^2[S]/4^2[F] resonances would likely involve motion localized on the planar monomer since there is little charge-transfer character of either 8^2[S] or 4^2[F] resonances.

	Anion	5^2[F]/3^2[F]	7^2[S]/5^2[F]
Tilt (P, N)	~3, ~12	~2, ~2	~11, ~2
P(C=O)	1.24, 1.24	1.23, 1.23	1.21, 1.20
N(C=O)	1.20, 1.20	1.23, 1.23	1.22, 1.22
P(C=C)	1.37, 1.37	1.36, 1.49	1.38, 1.36
N(C=C)	1.32, 1.35	1.36, 1.37	1.36, 1.45

Table S1: Summary of conical intersection geometries. Geometrical parameters for carbonyl tilt in units of degrees and bond lengths in Å. P = planar monomer and N = non-planar monomer in the X^2A anion in Fig. 1b. In each column, the first set of values corresponds to the less substituted end/side of the monomer.

Selected carbonyl wagging modes

Illustration of several of the wagging modes included in Fig. 2c. The other wagging modes have similar displacements of the non-planar carbonyl group. The combined wagging and breathing vibration at 899 cm^{-1} (and perhaps also 704 cm^{-1}) is the main Franck-Condon active mode responsible for the vibrational photoexcitation competition. In the illustrations, the left-hand monomer has carbonyl groups in the *para*-quinone ring plane, while the right-hand monomer has the carbonyl groups bent out of the *para*-quinone ring plane.

573 cm^{-1} [0.07 eV]

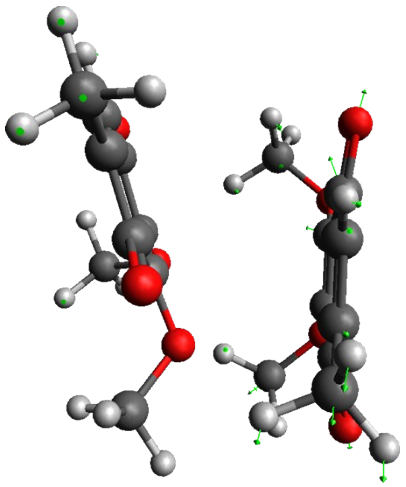

704 cm^{-1} [0.09]

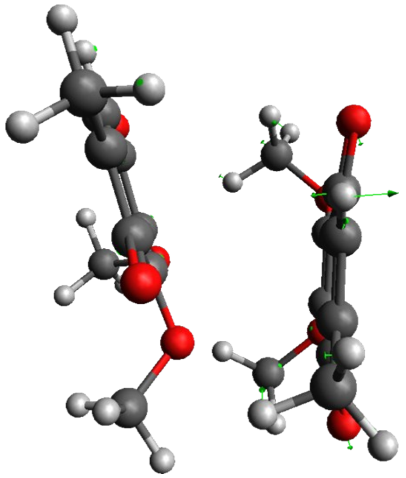

899 cm^{-1} [0.11 eV]

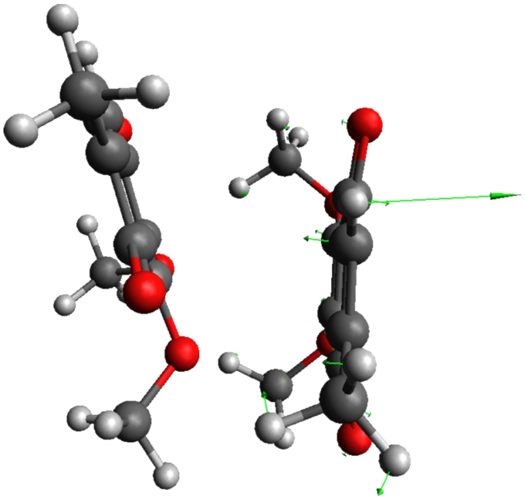

References

1. Bull, J. N.; West, C. W.; Verlet, J. R. R. *Phys. Chem. Chem. Phys.* **2015,** *17,* 16125.
2. Bull, J. N.; West, C. W.; Verlet, J. R. R. *Chem. Sci.* **2015,** *6,* 1578.
3. West, C. W.; Bull, J. N.; Antonkov, E.; Verlet, J. R. R. *J. Phys. Chem. A* **2014,** *118,* 11346.
4. Horke, D. A.; Li, Q.; Blancafort, L.; Verlet, J. R. R. *Nat. Chem.* **2013,** *5,* 711.
5. Eppink, A. T. J. B.; Parker, D. H. *Rev. Sci. Instrum.* **1997,** *68,* 3477-3484.
6. Horke, D. A.; Roberts, G. M.; Lecointre, J.; Verlet, J. R. R. *Rev. Sci. Instrum.* **2012,** *83,* 063101.
7. Roberts, G. M.; Nixon, J. L.; Lecointre, J.; Wrede, E.; Verlet, J. R. R. *Rev. Sci. Instrum.* **2009,** *80,* 053104.
8. Zare, R. N. *Mol. Photochem.* **1972,** *4,* 1.
9. Bull, J. N.; West, C. W.; Verlet, J. R. R. *Phys. Chem. Chem. Phys.* **2015,** submitted.
10. Amrein, A.; Simpson, R.; Hackett, P. *J. Chem. Phys.* **1991,** *95,* 1781.
11. Pinare, J. C.; Baguenard, B.; Bordas, C.; Broyer, M. *Phys. Rev. Lett.* **1998,** *81,* 2225.
12. Climen, B.; Pagliarulo, F.; Ollagnier, A.; Baguenard, B.; Concina, B.; Lebeault, M. A.; Lepine, F.; Bordas, C. *Eur. Phys. J. D* **2007,** *43,* 85.
13. Baguenard, B.; Pinare, J. C.; Bordas, C.; Broyer, M. *Phys. Rev. A* **2001,** *63,* 023204.
14. Frisch, M. J.; Trucks, G. W.; Schlegel, H. B.; Scuseria, G. E.; Robb, M. A.; Cheeseman, J. R.; Scalmani, G.; Barone, V.; Mennucci, B.; Petersson, G. A.; Nakatsuji, H.; Caricato, M.; Li, X.; Hratchian, H. P.; Izmaylov, A. F.; Bloino, J.; Zheng, G.; Sonnenberg, J. L.; Hada, M.; Ehara, M.; Toyota, K.; Fukuda, R.; Hasegawa, J.; Ishida, M.; Nakajima, T.; Honda, Y.; Kitao, O.; Nakai, H.; Vreven, T.; Montgomery, J. . J. A.; Peralta, J. E.; Ogliaro, F.; Bearpark, M.; Heyd, J. J.; Brothers, E.; Kudin, K. N.; Staroverov, V. N.; Kobayashi, R.; Normand, J.; Raghavachari, K.; Rendell, A.; Burant, J. C.; Iyengar, S. S.; Tomasi, J.; Cossi, M.; Rega, N.; Millam, N. J.; Klene, M.; Knox, J. E.; Cross, J. B.; Bakken, V.; Adamo, C.; Jaramillo, J.; Gomperts, R.; Stratmann, R. E.; Yazyev, O.; Austin, A. J.; Cammi, R.; Pomelli, C.; Ochterski, J. W.; Martin, R. L.; Morokuma, K.; Zakrzewski, V. G.; Voth, G. A.; Salvador, P.; Dannenberg, J. J.; Dapprich, S.; Daniels, A. D.; Farkas, Ö.; Foresman, J. B.; Ortiz, J. V.; Cioslowski, J.; Fox, D. J. *Gaussian, Inc.;* Tech. rep.; Wallingford CT, 2009.
15. Schmidt, M. W.; Baldridge, K. K.; Boatz, J. A.; Elbert, S. T.; Gordon, M. S.; Jensen, J. H.; Koseki, S.; Matsunaga, N.; Nguyen, K. A.; Su, S.; Windus, T. L.; Dupuis, M.; Montgomery, J. A. *J. Comput. Chem.* **1993,** *14,* 1347.
16. Stewart, J. J. P. *J. Mol. Model.* **2007,** *13,* 1173.
17. Chai, J.-D.; Head-Gordon, M. *Phys. Chem. Chem. Phys.* **2008,** *10,* 6615.
18. Thanthiriwatte, K. S.; Hohenstein, E. G.; Burns, L. A.; Sherrill, C. D. *J. Chem. Theor. Comp.* **2011,** *7,* 88.
19. DiLabio, G. A.; Johnson, E. R.; Otero-de-la-Roza, A. *Phys. Chem. Chem. Phys.* **2013,** *15,* 12821.
20. Li, A.; Muddana, H. S.; Gilson, M. K. *J. Chem. Theory Comput.* **2015,** *10,* 1563.
21. Dunning Jr., T. H. *J. Chem. Phys.* **1989,** *90,* 1007.
22. Boys, S. F.; Bernardi, F. *Mol. Phys.* **1970,** *19,* 553.
23. Granovsky, A. A. *J. Chem. Phys.* **2011,** *134,* 214113.
24. Bearpark, M. J.; Robb, M. A.; Schlegel, H. B. *Chem. Phys. Lett.* **1994,** *223,* 269.
25. Skurski, P.; Gutowski, M.; Simons, J. *Int. J. Quant. Chem.* **2000,** *80,* 1024.
26. Frisch, M. J.; Pople, J. A.; Binkley, J. S. *J. Chem. Phys.* **1984,** *80,* 3265.
27. Jordan, K. D.; Wang, F. *Annu. Rev. Phys. Chem.* **2003,** *54,* 367.
28. Hammer, N. I.; Diri, K.; Jordan, K. D.; Desfrançois, C.; Compton, R. N. *J. Chem. Phys.* **2003,** *119,* 3650.
29. Sanov, A. *Annu. Rev. Phys. Chem.* **2014,** *65,* 341.
30. Wigner, E. P. *Phys. Rev.* **1948,** *73,* 1002.
31. Reed, K. J.; Zimmerman, A. H.; Andersen, H. C.; Brauman, J. I. *J. Chem. Phys.* **1976,** *64,* 1368.
32. Cederbaum, L. S.; Friedman, R. S.; Ryaboy, V. M.; Moiseyev, N. *Phys. Rev. Lett.* **2003,** *90,* 013001.

All frequency-resolved photoelectron spectra

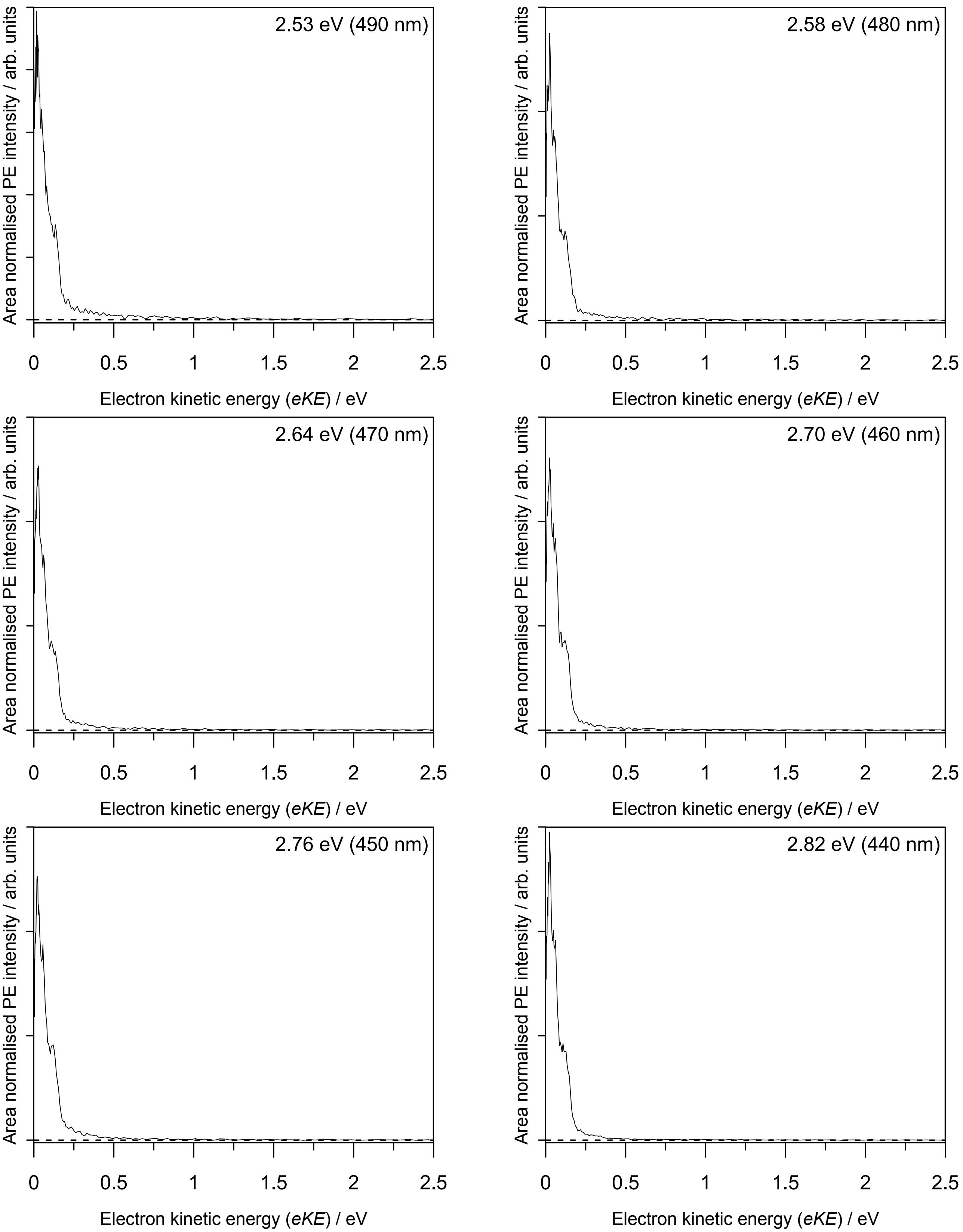

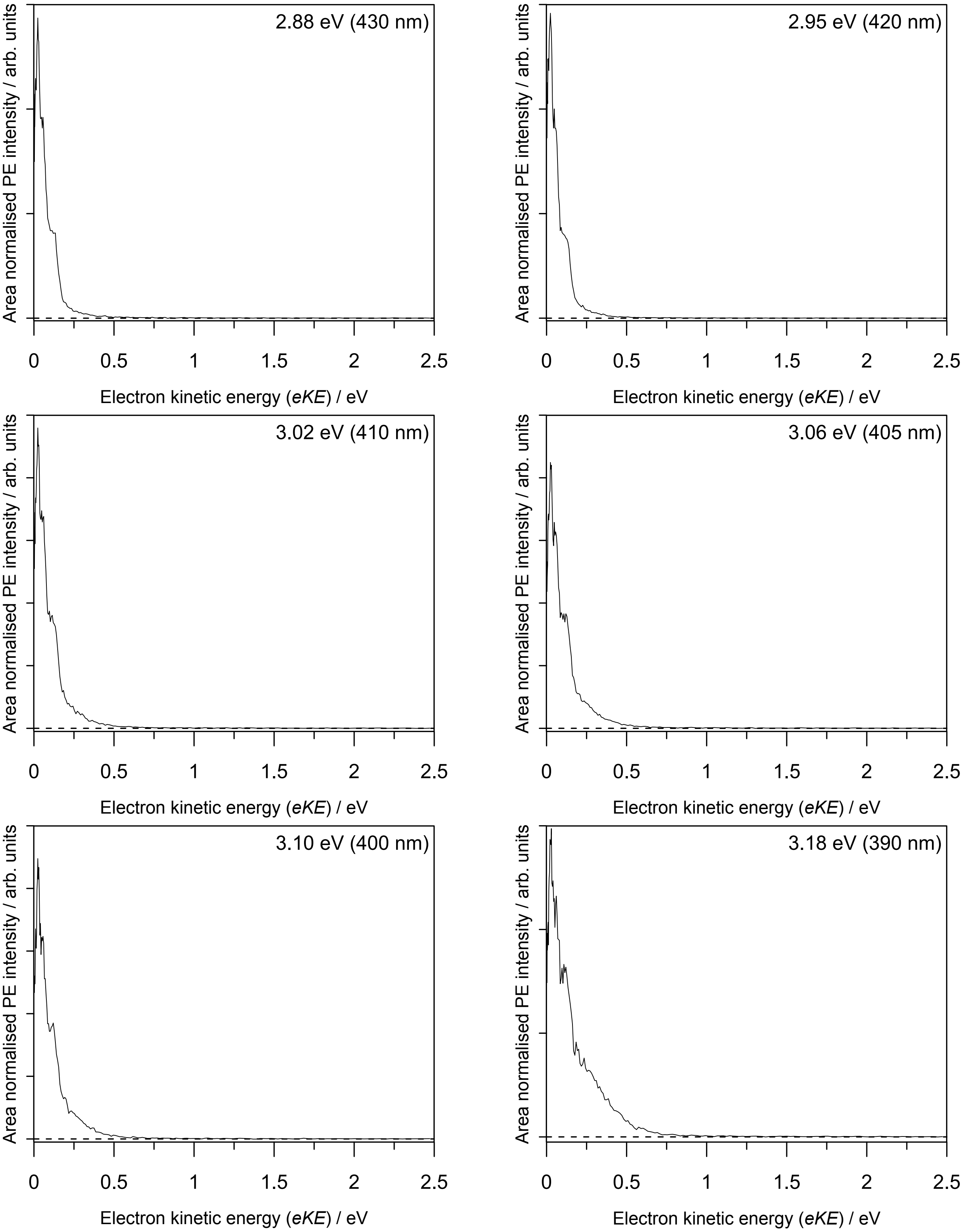
2.88 eV (430 nm)
2.95 eV (420 nm)
3.02 eV (410 nm)
3.06 eV (405 nm)
3.10 eV (400 nm)
3.18 eV (390 nm)
Area normalised PE intensity / arb. units
Electron kinetic energy (eKE) / eV
0
0.5
1
1.5
2
2.5

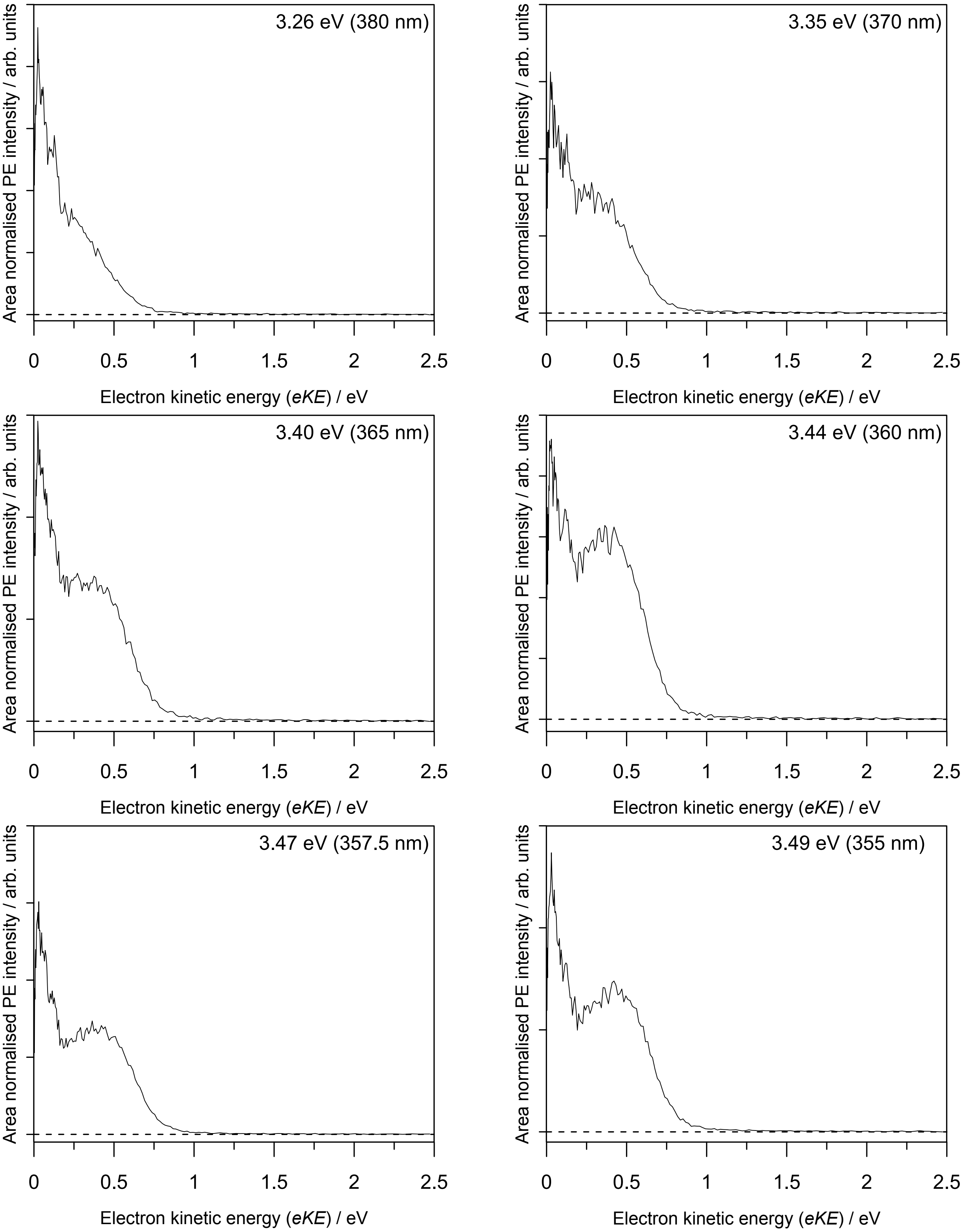
3.26 eV (380 nm)
3.35 eV (370 nm)
3.40 eV (365 nm)
3.44 eV (360 nm)
3.47 eV (357.5 nm)
3.49 eV (355 nm)
Area normalised PE intensity / arb. units
0
0.5
1
1.5
2
2.5
Electron kinetic energy (*eKE*) / eV

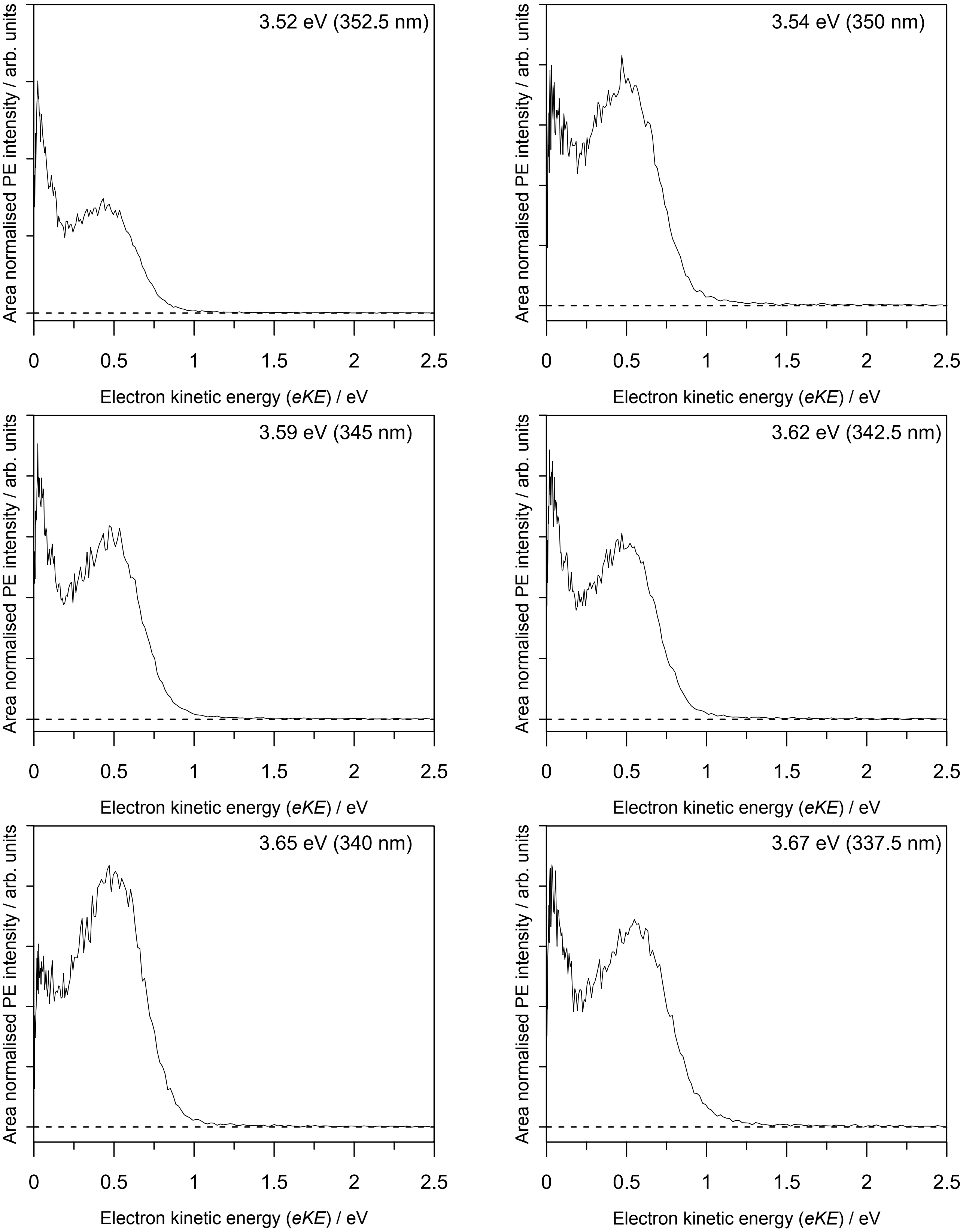

3.52 eV (352.5 nm)
3.54 eV (350 nm)
3.59 eV (345 nm)
3.62 eV (342.5 nm)
3.65 eV (340 nm)
3.67 eV (337.5 nm)
Area normalised PE intensity / arb. units
Electron kinetic energy (eKE) / eV
0
0.5
1
1.5
2
2.5

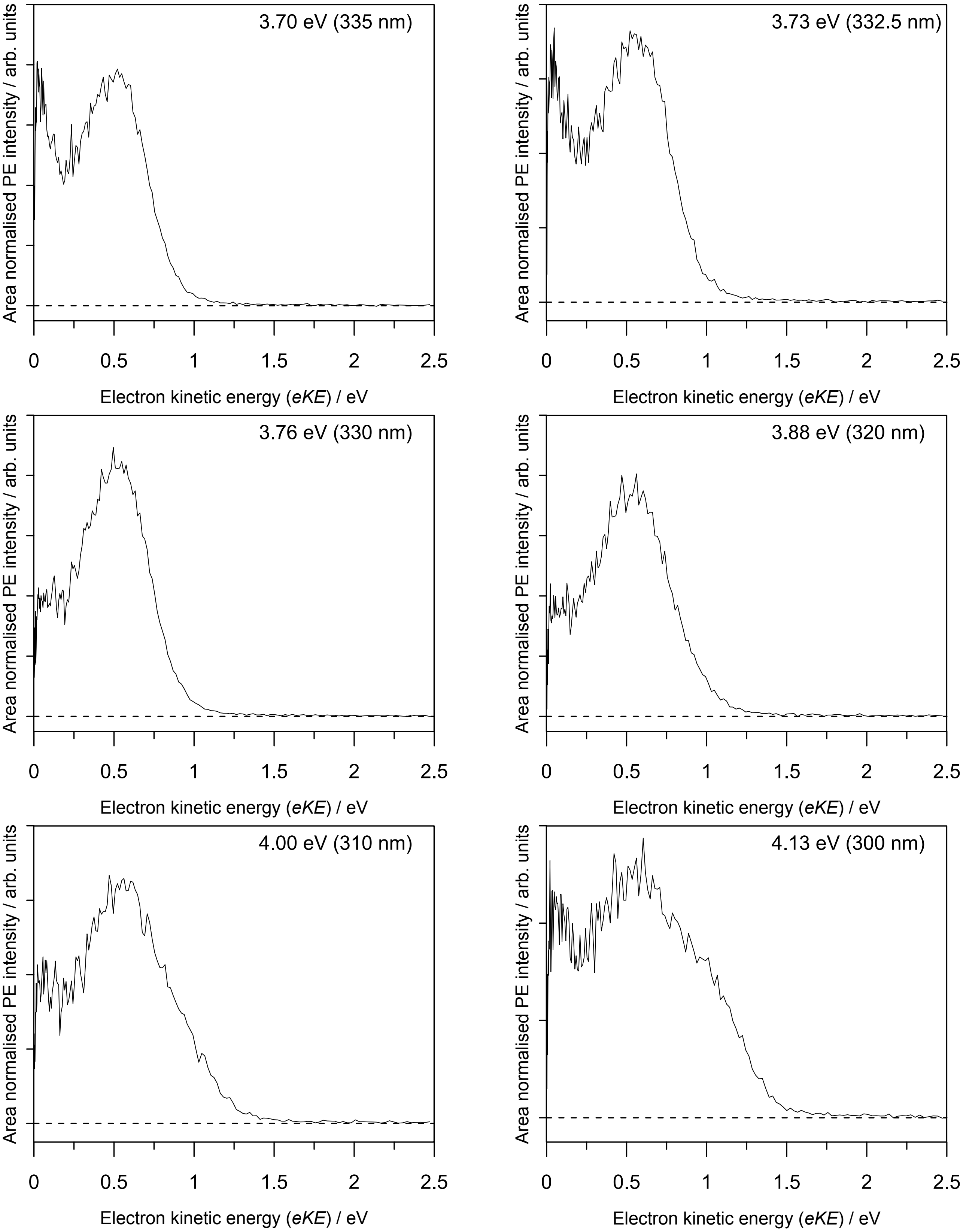
3.70 eV (335 nm)
3.73 eV (332.5 nm)
3.76 eV (330 nm)
3.88 eV (320 nm)
4.00 eV (310 nm)
4.13 eV (300 nm)
Area normalised PE intensity / arb. units
Electron kinetic energy (eKE) / eV
0
0.5
1
1.5
2
2.5

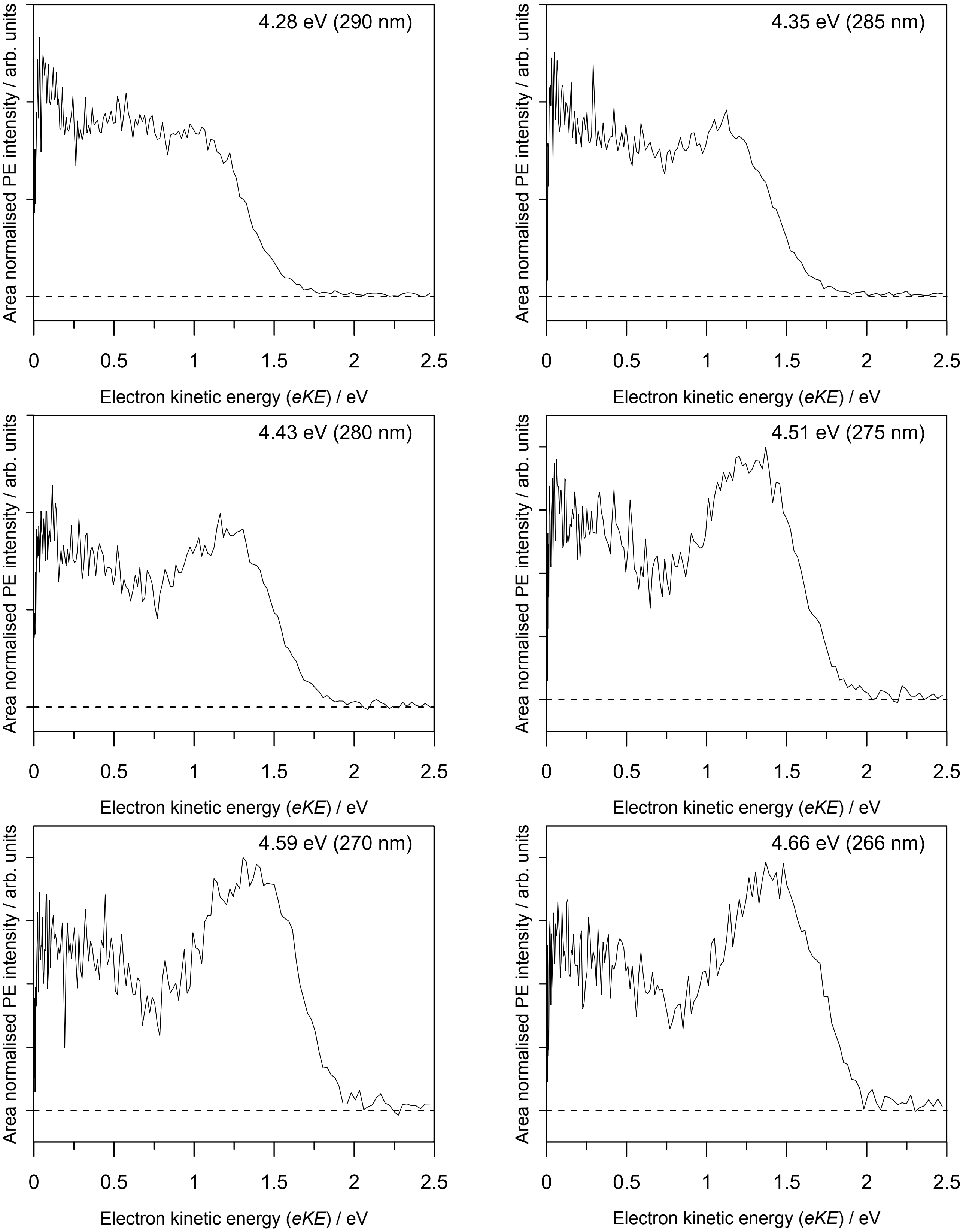
4.28 eV (290 nm)
4.35 eV (285 nm)
4.43 eV (280 nm)
4.51 eV (275 nm)
4.59 eV (270 nm)
4.66 eV (266 nm)
Area normalised PE intensity / arb. units
Electron kinetic energy (eKE) / eV
0
0.5
1
1.5
2
2.5

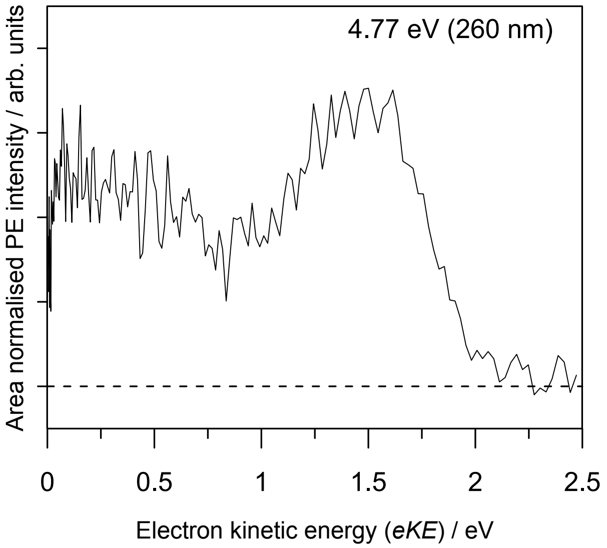

4.77 eV (260 nm)
Area normalised PE intensity / arb. units
0
0.5
1
1.5
2
2.5
Electron kinetic energy (eKE) / eV
